# Disease-Associated *Streptococcus pneumoniae* Genetic Variation

**DOI:** 10.3201/eid3001.221927

**Published:** 2024-01

**Authors:** Shimin Yang, Jianyu Chen, Jinjian Fu, Jiayin Huang, Ting Li, Zhenjiang Yao, Xiaohua Ye

**Affiliations:** Guangdong Pharmaceutical University, Guangzhou, China (S. Yang, J. Chen, J. Huang, T. Li, Z. Yao, X. Ye);; Liuzhou Maternity and Child Health Care Hospital, Liuzhou, China (J. Fu)

**Keywords:** Streptococcus pneumoniae, genome-wide association study, bacterial genomes, pathogenicity, bacteria, China

## Abstract

*Streptococcus pneumoniae* is an opportunistic pathogen that causes substantial illness and death among children worldwide. The genetic backgrounds of pneumococci that cause infection versus asymptomatic carriage vary substantially. To determine the evolutionary mechanisms of opportunistic pathogenicity, we conducted a genomic surveillance study in China. We collected 783 *S. pneumoniae* isolates from infected and asymptomatic children. By using a 2-stage genomewide association study process, we compared genomic differences between infection and carriage isolates to address genomic variation associated with pathogenicity. We identified 8 consensus k-mers associated with adherence, antimicrobial resistance, and immune modulation, which were unevenly distributed in the infection isolates. Classification accuracy of the best k-mer predictor for *S. pneumoniae* infection was good, giving a simple target for predicting pathogenic isolates. Our findings suggest that *S. pneumoniae* pathogenicity is complex and multifactorial, and we provide genetic evidence for precise targeted interventions.

*Streptococcus pneumoniae* is a pathogen that causes community-associated infections in young children <5 years of age (*1*,*2*). It can asymptomatically colonize the nasopharynx and upper airway in healthy children (up to 60%) and can also invade sterile sites and lead to infections from mild to life-threatening, which can result in substantial illness and death worldwide (*1*,*3*,*4*). Despite the widespread use of pneumococcal vaccines to immunize children, *S. pneumoniae* remains the leading cause of life-threatening diseases. Worldwide, the increasing disease burden of *S. pneumoniae* is alarming; an estimated 1 million children <5 years of age die of pneumococcal disease every year (*5*). All pneumococcal diseases arise from bacterial colonization, and the adaptability of the virulence characteristics enhances pneumococcal persistence in colonization of the host respiratory tract, suggesting that nasopharyngeal carriage of *S. pneumoniae* plays a key role in development and transmission of pneumococcal diseases (*6*). Pneumococcal disease is one of the most common infectious diseases caused by asymptomatic *S. pneumoniae* colonization in humans. Eliminating this opportunistic pathogenic bacterium requires knowledge of the pathogenicity-associated genetic elements that distinguish infection from carriage isolates. Previous studies have been limited to exploring virulence factors and molecular characterization of invasive *S. pneumoniae* isolates (*7*,*8*).

Whole-genome sequencing (WGS) has become a powerful tool for bacterial genotyping; costs have been decreasing as accessibility increases. The high-dimensional genomic data can provide unprecedented resolution for identifying subtle genomic variations. Genomewide association studies (GWAS) are increasingly used to detect novel genes and genetic elements associated with bacterial phenotypes, which may provide insight for future preventive strategies and control measures (*9*–*12*). In brief, traditional GWAS methods can be used to identify large numbers of common genetic variants, usually single-nucleotide polymorphisms (SNPs), to determine the genetic basis of bacterial phenotypes of interest. However, considering the high genomic plasticity of many species of bacteria, traditional GWAS methods can only partially identify the phenotype-associated genetic variants. To avoid the limitations of SNP-based GWAS, we used k-mers (DNA words of length k) as an alternative method, which can capture different types of variants (*13*,*14*).

To determine whether genetic variation is unevenly enriched in *S. pneumoniae* infection isolates, we used multiple GWAS analyses to compare genomic differences between infection and carriage isolates. Study protocols were approved by the Ethics Committee of Guangdong Pharmaceutical University (2019–19) and the Ethics Committee of Liuzhou Maternity and Child Healthcare Hospital (2018–84). We obtained written informed consent from parents or legal guardians on behalf of the children.

## Methods

### Sampling

During 2015–2021, we collected clinical samples from infected children and nasal swab samples from healthy children in southern China (Guangxi and Guangdong Provinces). From hospitalized infected children, we collected 349 nonrepetitive pneumococcal isolates (e.g., blood, bronchoalveolar lavage fluid, sputum, middle ear fluid), of which 342 were noninvasive and 7 invasive. The eligibility criteria for infected children were having clinical infectious manifestations such as cough, respiratory secretions, abnormal lung sounds, dyspnea, or fever >38°C, with or without infiltrates seen on chest radiographs; having *S. pneumoniae* infection diagnosed by clinical doctors on the basis of signs and symptoms; and having *S. pneumoniae* isolated from clinical infection sites. In terms of asymptomatic carriage isolates, we sampled 434 isolates from healthy children in kindergarten.

### Whole-Genome Sequencing 

We performed high-throughput genome sequencing on a Hiseq 2000 machine (Illumina, https://www.illumina.com) to obtain paired-end 150-bp reads. We assessed the quality of the raw sequenced reads by using FastQC version 0.11.5 (https://github.com/s-andrews/FastQC) and trimmed for low quality reads and adaptor regions by using Trimmomatic version 0.36 (https://github.com/usadellab/Trimmomatic). We then assembled trimmed reads by using SPAdes version 3.6.1 (https://github.com/ablab/spades). We used PathogenWatch (https://pathogen.watch) to predict global pneumococcal sequencing cluster (GPSC), multilocus sequence typing (MLST), and serotyping for all genomes.

### Phylogenetic Analyses

To generate the variant sites with SNPs, we mapped assembled contigs to a standard reference genome *S. pneumoniae* R6 by using Snippy version 4.4.5 (https://github.com/tseemann/snippy). We used the generated core SNP alignment to construct a maximum-likelihood phylogenetic tree by using the generalized time reversible plus gamma model and 100 bootstrap replicates with FastTree version 2.1.10 (http://www.microbesonline.org/fasttree). We visualized and annotated the phylogenetic tree by using ChiPlot (https://www.chiplot.online).

### Counting and Annotating k-mers

We scanned all k-mers that were 9- to 100-bp long from all assembled reads by using fsm-lite (https://github.com/nvalimak/fsm-lite) and filtered them to obtain 10,591,337 k-mers seen on 1%–99% of the total samples. To identify the relevant genes by using BWA-MEM (the Burrows-Wheeler Aligner with maximal exact matches alignment tool, https://github.com/lh3/bwa), we mapped all k-mers to 10 *S. pneumoniae* reference genomes (CGSP14, D39, Hungary^19A^-6, R6, Taiwan^19F^-14, TIGR4, Spain^23F^-ST81, ATCC 49619, EF3030, and MDRSPN001) obtained from the Virulence Factor Database (http://www.mgc.ac.cn/VFs) and previous studies. We determined gene ontology annotations by using the UniProt (https://beta.uniprot.org).

### Multiple GWAS Analyses of Disease-Associated k-mers

To explore the genomewide associations between genetic elements (k-mers) and *S. pneumoniae* disease status (infection or carriage), and thus to identify infection-associated k-mers, we used GWAS methods. Because of the high-dimensional genomic data structures, we used multiple GWAS methods: the linear mixed model (LMM; (https://github.com/mgalardini/pyseer), phylogenetic-based approach (Scoary; https://github.com/AdmiralenOla/Scoary), variable selection using random forests (VSURF; https://github.com/robingenuer/VSURF), and least absolute shrinkage and selection operator (LASSO; https://scikit-learn.org/stable/modules/generated/sklearn.linear_model.Lasso.html) regression.

**Figure 1:**
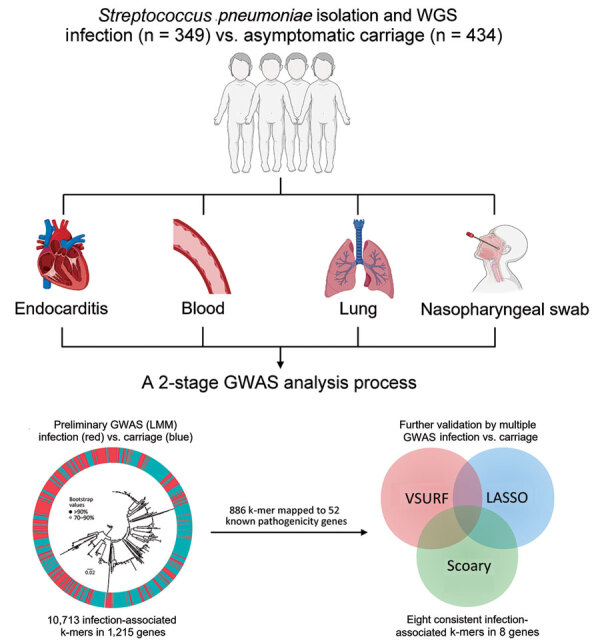
Two-stage GWAS analysis process used to detect infection-associated *Streptococcus pneumoniae* k-mers in study of disease-associated *Streptococcus pneumoniae* genetic variation. GWAS, genome-wide association studies; LASSO, least absolute shrinkage and selection operator; LMM, linear mixed model; VSURF, variable selection using random forests; WGS, whole-genome sequencing.

In brief, we used a 2-stage analysis process to detect the infection-associated k-mers by comprehensive GWAS analyses (Figure 1). First, we fitted a univariate LMM to initially screen infection-associated k-mers by using the Pyseer tool (version 1.3.10) (*15*). To correct for the population structure, we used the similarity pyseer command of Pyseer, which computes a similarity kinship matrix on the basis of the core genome SNPs. For covariates, the GWAS analysis used host age (years) and sex. Second, we used multiple methods (Scoary, LASSO, and VSURF) to minimize false-positive associations and identify consensus infection-associated k-mers by Venn diagram. In the GWAS analyses, we used the Bonferroni correction (α/*N*) to control for false-positive rates resulting from multiple comparisons of 1,418,815 k-mers (adjusted p value threshold 3.52 × 10^−8^). Scoary is an ultrafast software tool for GWAS analyses that uses a phylogenetic-based method to adjust population structure. The LASSO regression is suitable for high-dimensional data structures, and the coefficients of nonrelevant variables can be compressed to zero to solve the problem of model overfitting (*16*). We used VSURF, based on random forest (RF), to perform a 2-step feature selection on the variables (*17*). Initially, VSURF ranks the variables according to the importance measure by using the RF permutation-based score of importance to obtain a subset of important variables, and then it uses a stepwise forward strategy for variable introduction based on the smallest out-of-bag error. More precisely, a variable is added only if the error decrease is larger than a threshold. We ranked the importance of k-mers by the mean decrease in impurity (mean decrease Gini), which is a measure of the predictor’s contribution to the correct sample classification. We compiled associated phenotype data for all 783 isolates (Appendix Table 1) and deposited sequences in the National Center for Biotechnology Information Sequence Read Archive database (https://www.ncbi.nlm.nih.gov/sra; projection no. PRJNA976286). The k-mer sequences and output results files from several GWAS analyses are publicly available (https://doi.org/10.6084/m9.figshare.24466606.v3).

## Results

### Characteristics of *S. pneumoniae* Isolates

Of the 349 children with *S. pneumoniae* infection, 342 (98.0%) had noninvasive disease (264 pneumonia, 49 bronchitis, 13 otitis media, 9 upper respiratory infection, 6 nasosinusitis, and 1 corneal ulcer), and 7 (2.0%) had invasive disease (6 bacteremia and 1 endocarditis). χ^2^ test results indicated no differences between infection and carriage isolates with regard to host sex (p = 0.359) but significant differences with regard to age (p<0.001) (Appendix Table 2).

### Association between Genotypes and Disease Status

**Figure 2:**
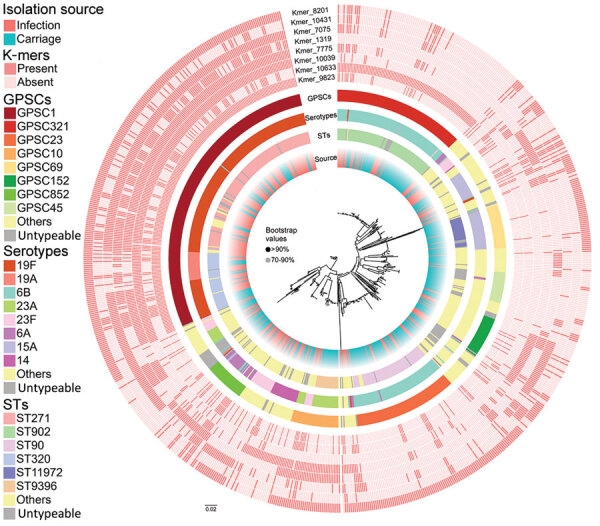
Whole-genome phylogenetic tree showing genetic similarity of 783 *Streptococcus pneumoniae* isolates in a study of disease-associated *Streptococcus pneumoniae* genetic variation. The colored strips at the tips of the tree (from inner to outer) represent isolate metadata (source, STs, serotypes, and GPSCs) and infection-associated k-mers found in the final model. GPSC, global pneumococcal sequencing cluster; ST, sequence type.

The most prevalent GPSCs for infection isolates were GPSC1 (45.9%), GPSC321 (9.2%), and GPSC852 (5.4%); the predominant GPSCs for carriage isolates were GPSC321 (16.1%), GPSC1 (15.4%), and GPSC23 (15.0%). In terms of sequence types (STs), the most common genotypes for infection isolates were ST271 (29.2%), ST320 (9.5%), and ST902 (7.2%); the predominant genotypes for carriage isolates were ST902 (15.9%), ST90 (13.8%), and ST271 (8.5%). The most prevalent serotypes for infection isolates were 19F (43.0%), 6B (15.2%), and 23F (8.3%); and the predominant serotypes for carriage isolates were 6B (32.7%), 19F (13.1%), and 15A (11.1%). The results indicated potential genotype differences between infection and carriage isolates. In addition, the phylogenetic tree based on core SNPs revealed that several genotypes (GPSCs/STs/serotypes) from infection and carriage isolates clustered in the same branches (Figure 2). Moreover, we found statistically significant differences in the proportion of specific GPSCs/STs/serotypes between infection and carriage isolates (Table 1), indicating that these isolates are associated with infection.

**Table 1:** Association analysis between dominant genotypes and disease status from study of disease-associated *Streptococcus pneumoniae* genetic variation*

Genotype	Infection isolates, no. (%), n = 349	Carriage Isolates, no. (%), n = 434	χ^2^	p value	OR (95% CI)
ST					
ST271	102 (29.2)	37 (8.5)	56.78	**<0.001**	4.43 (2.95–6.67)
ST902	25 (7.2)	69 (15.9)	13.97	**<0.001**	0.41 (0.25–0.66)
ST90	13 (3.7)	60 (13.8)	23.34	**<0.001**	0.24 (0.13–0.45)
ST320	33 (9.5)	24 (5.5)	4.42	**0.036**	1.78 (1.03–3.08)
ST11972	3 (0.9)	24 (5.5)	12.67	**<0.001**	0.15 (0.04–0.50)
ST9396	1 (0.3)	22 (5.1)	15.52	**<0.001**	0.05 (0.01–0.40)
Serotype					
19F	150 (43.0)	57 (13.1)	88.61	**<0.001**	4.99 (3.51–7.08)
6B	53 (15.2)	142 (32.7)	31.80	**<0.001**	0.37 (0.26–0.53)
15A	12 (3.4)	48 (11.1)	15.88	**<0.001**	0.29 (0.15–0.55)
23F	29 (8.3)	21 (4.8)	3.90	0.056	1.78 (0.96–3.35)
23A	10 (2.9)	32 (7.4)	7.74	**0.005**	0.37 (0.18–0.77)
6A	24 (6.9)	10 (2.3)	9.74	**0.002**	3.13 (1.48–6.64)
19A	15 (4.3)	11 (2.5)	1.87	0.171	1.73 (0.78–3.81)
14	15 (4.3)	11 (2.5)	1.87	0.171	1.73 (0.78–3.81)
GPSC					
GPSC1	160 (45.9)	67 (15.4)	88.88	**<0.001**	4.64 (3.32–6.48)
GPSC321	32 (9.2)	70 (16.1)	8.27	**0.004**	0.52 (0.34–0.82)
GPSC23	15 (4.3)	65 (15.0)	24.05	**<0.001**	0.25 (0.14–0.45)
GPSC10	9 (2.6)	28 (6.5)	6.44	**0.011**	0.38 (0.18–0.81)
GPSC69	3 (0.9)	31 (7.1)	18.39	**<0.001**	0.11 (0.04–0.35)
GPSC152	13 (3.7)	18 (4.2)	0.09	0.763	0.89 (0.44–1.83)
GPSC852	19 (5.4)	12 (2.8)	3.65	0.056	2.02 (0.98–4.17)

*Boldface indicates statistical significance. GPSC, global pneumococcal sequencing cluster; OR, odds ratio; ST, sequence type.

### Preliminary Screening for Infection-Associated k-mers by LMM

**Figure 3:**
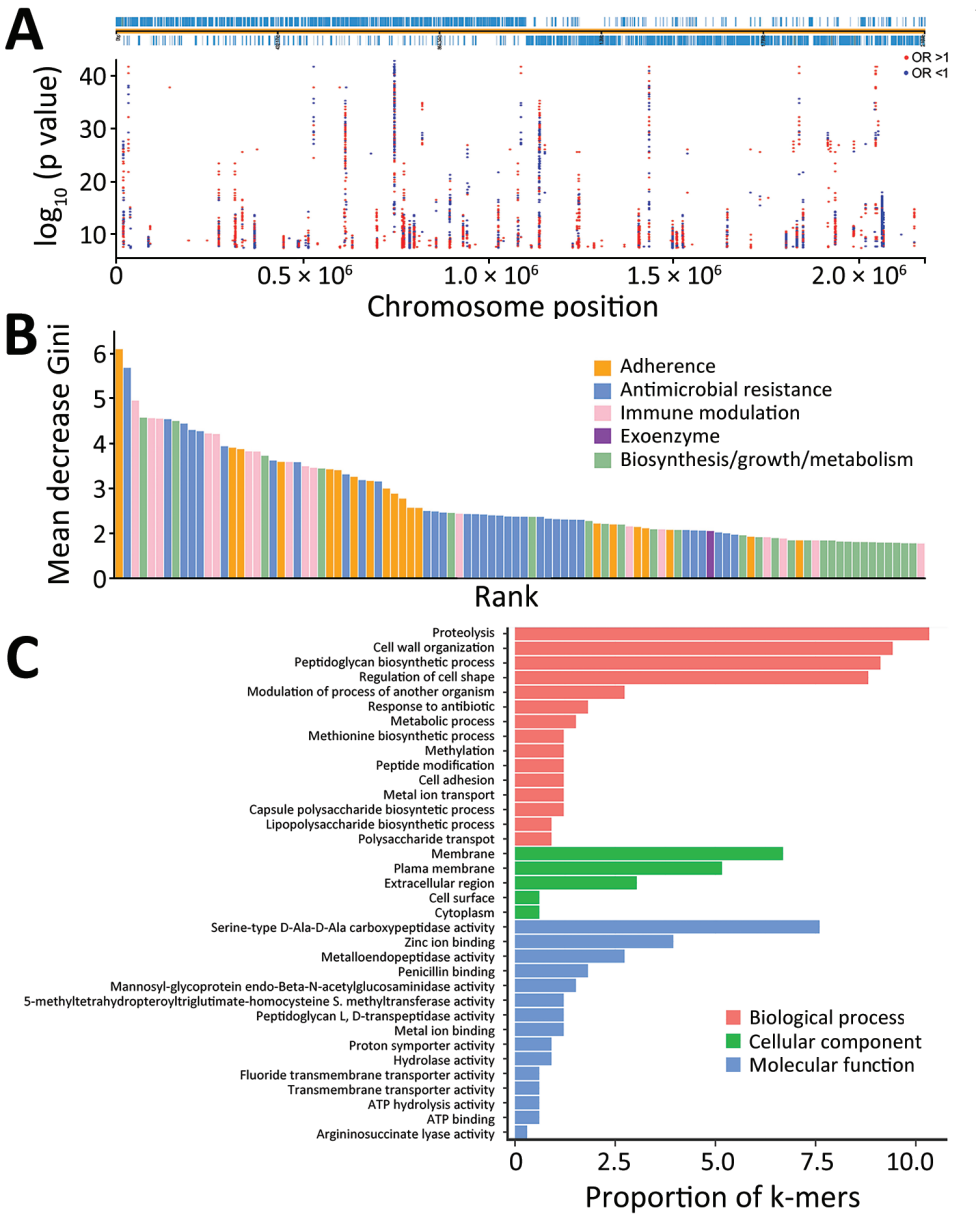
Preliminary screening for infection-associated k-mers by linear mixed model in study of disease-associated *Streptococcus pneumoniae* genetic variation. A) Manhattan plot showing statistical significance and chromosomal location of k-mers mapped to a complete reference genome (TIGR4; GenBank accession no. NC_003028.3). B) Importance of the top 100 k-mer predictors in a simpler model with 886 k-mers. C) Gene ontology annotations of the top 100 k-mer predictors. OR, odds ratio.

We identified 10,591,337 k-mers from the assemblies of 783 *S. pneumoniae* isolates and then filtered out low-frequency k-mers for a reduced matrix with 1,418,815 k-mers. Using those k-mers for GWAS, we performed a univariate LMM analysis to initially identify 22,790 infection-associated k-mers; 10,713 k-mers were successfully mapped to 1,215 unique genes (Figure 3, panel A; Appendix Figure 1). In the initial model with 10,713 k-mers, we used the RF model to assess the prediction effect. The classification balanced accuracy based on cross-validation was 93.60% (95% CI 91.48%–95.72%) (Table 2); the area under the curve (AUC), based on the out-of-bag risk scores of the classifier, was 0.98. In the LMM analysis, the QQ-plot indicated that population structure was well controlled at low p values (p<0.01) (Appendix Figure 2). Because of the considerable redundancy among the genetic elements in risk prediction, studying all k-mer combinations had little benefit; therefore, we used a simpler model with 886 k-mers successfully mapped to 52 antibiotic resistance or virulence genes (Appendix Table 3). The classification balanced accuracy was 91.28% (95% CI 89.34%–93.22%) (Table 2); the AUC was 0.96, suggesting that the power of these 886 k-mers for predicting disease status was close to that of the model with 10,713 k-mers.

**Table 2:** Resubstitution estimate and cross-validation results based on random forest models used in study of disease-associated *Streptococcus pneumoniae* genetic variation*

Evaluation indicators	10,713 k-mer predictors			886 k-mer predictors			8 k-mer predictors	
	Resubstitution estimate	10-fold cross-validation estimate		Resubstitution estimate	10-fold cross-validation estimate		Resubstitution estimate	10-fold cross-validation estimate
Accuracy	98.60	93.23		96.42	90.81		90.93	90.04
Balanced accuracy	98.65	93.60		96.61	91.28		91.48	90.89
Sensitivity	99.13	94.48		97.91	92.87		94.27	93.72
Specificity	98.18	92.71		95.31	89.69		88.70	88.07
PPV	97.71	90.27		93.98	86.27		84.81	83.65
NPV	99.31	95.63		98.39	94.48		95.85	95.18
Kappa	0.97	0.86		0.93	0.81		0.81	0.80

*Values are percentages except for kappa, which is reported as a value ranging from –1 to 1. NPV, negative predictive value; PPV, positive predicitive value.

In addition, we sorted the 886 disease-associated k-mers according to estimated importance (Figure 3, panel B). The k-mers were mainly associated with antimicrobial resistance (34%), adherence (20%), immune modulation (17%), and exoenzyme (1%). Moreover, the k-mers were divided into 3 functional gene ontology categories. Among those categories, proteolysis and cell wall organization were the largest subcategories in the biological process, membrane was the most enriched term in the cellular component, and serine-type D-Ala-D-Ala carboxypeptidase activity was the top term in the molecular function (Figure 3, panel C).

### Further Validation of Infection-Associated k-mers by Multiple GWAS Analyses

**Figure 4:**
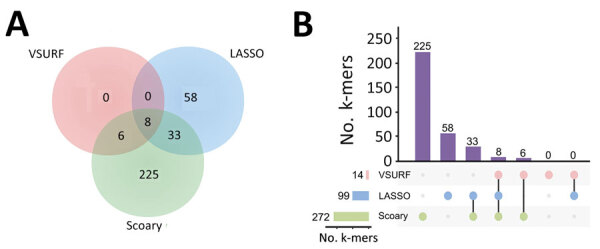
Further validation of infection-associated k-mers by multiple GWAS analyses in study of disease-associated *Streptococcus pneumoniae* genetic variation. A) Venn diagram visualization of the k-mers identified by 3 methods. B) UpSet plot visualization of the k-mers identified by 3 methods. LASSO, least absolute shrinkage and selection operator; VSURF, variable selection using random forests.

**Figure 5:**
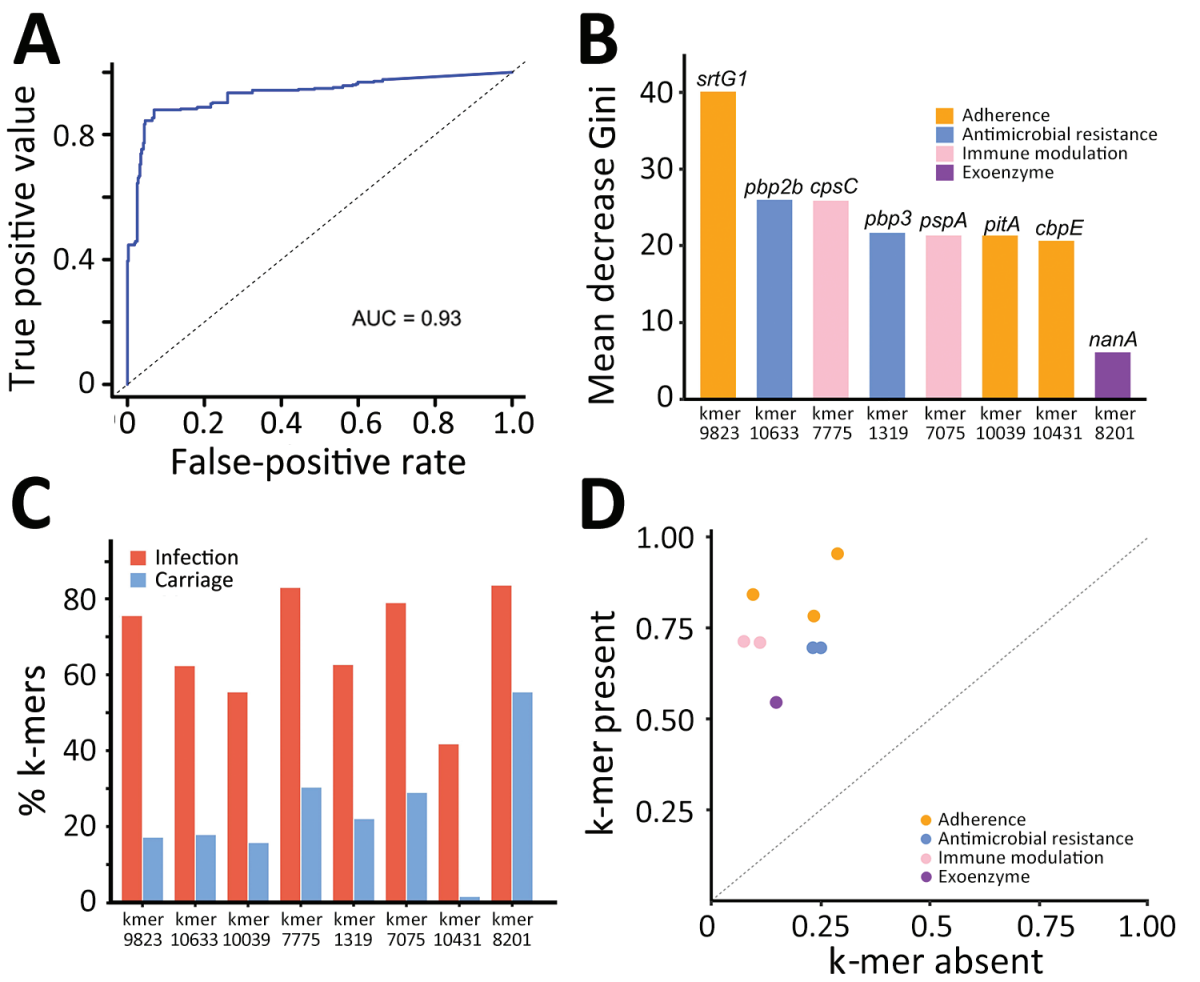
Prediction effect of the 8 k-mers identified in the final model used in study of disease-associated *Streptococcus pneumoniae* genetic variation. A) Receiver operating characteristic curve of the final model. B) Predictor importance of the 8 k-mers in the final model. C) Proportion of k-mer predictors between infection and carriage isolates. D) Change in risk score for a specific k-mer profile when the k-mer is present (y-axis) compared to absent (x-axis). AUC, area under the curve.

To reduce the complexity of the model, we used 3 methods to identify consensus infection-associated k-mers (Figure 4). On the basis of the 886 k-mers screened above, we observed consensus on genomewide statistically significant associations for pathogenicity k-mers; 8 k-mers were identified by all 3 methods. When we used the simplest model with the 8 k-mers, the classification balanced accuracy was 90.89% (95% CI 89.48%–92.31%) (Table 2), and the AUC value was 0.93 (Figure 5, panel A), suggesting that the power of the 8 k-mers to predict disease status was comparable to that of the model with 886 k-mers. Of note, the k-mer predictors still exhibited high classification balanced accuracy in the predominant GPSCs (95.34% for GPSC1 and 92.79% for GPSC321). The importance of the selected k-mers in the final model indicated that these predictors were mainly associated with adherence function (Figure 5, panel B). The highest ranked predictor (Kmer_9823 in sortase [*srtG1*]) achieved a classification accuracy of 79.57% on its own and also showed high classification accuracy in the predominant GPSCs (70.04% for GPSC1 and 85.29% for GPSC321). In addition, the best predictor (in *srtG1*) was associated with GPSC1 and GPSC321 (all p<0.05). For the additional validation analysis that used the best RF classifier k-mer (in *srtG1*), 2 independent datasets of *S. pneumoniae* genomes with genotype distribution similar to that of our study were available on the National Center for Biotechnology Information Assembly database (https://www.ncbi.nlm.nih.gov/assembly (data1: 60 noninvasive vs. 60 carriage isolates; data2: 60 invasive versus 60 carriage isolates; the prevalence of the predominant GPSCs [GPSC1 and GPSC321] was 58.3% for noninvasive, 55.0% for invasive and 30.0% for carriage isolates) (Appendix Table 4). Classification accuracy was 75.83% for data1 and 74.17% for data2, similar to that in the larger primary dataset in our study.

The proportion of k-mers differed significantly between infection and carriage isolates (all p<0.05) (Figure 5, panel C), indicating that the proportion of k-mers was substantially higher in infection isolates than in carriage isolates. The effect of each k-mer on the estimated risk score (Figure 5, panel D), indicated by a point above the diagonal, indicates that the risk score is increased when the k-mer profile is present. The presence of k-mers associated with adherence genes markedly increased the risk for *S. pneumoniae* infection (odds ratio [OR] 1.88 for Kmer_9823, OR 1.65 for Kmer_10039, and OR 1.69 for Kmer_10431) (Table 3).

**Table 3:** Association analysis between k-mers and disease status used in study of disease-associated *Streptococcus pneumoniae* genetic variation*

k-mer	Genes	Infection isolates, no. (%), n = 349	Carriage isolates, no. (%), n = 434	p value	OR (95%CI)
Kmer_9823	*srtG1*	264 (75.6)	75 (17.3)	8.55 × 10^–45^	1.88 (1.79–1.96)
Kmer_10633	*pbp2b*	218 (62.5)	78 (18.0)	2.68 × 10^–37^	1.77 (1.71–1.84)
Kmer_10039	*pitA*	194 (55.6)	69 (15.9)	1.47 × 10^–31^	1.65 (1.51–1.79)
Kmer_7775	*cpsC*	290 (83.1)	132 (30.4)	6.59 × 10^–49^	1.75 (1.66–1.83)
Kmer_1319	*pbp3*	219 (62.8)	96 (22.1)	9.95 × 10^–31^	1.79 (1.70–1.87)
Kmer_7075	*pspA*	276 (79.1)	126 (29.0)	4.31 × 10^–44^	1.64 (1.55–1.72)
Kmer_10431	*cbpE*	146 (41.8)	7 (1.6)	3.38 × 10^–45^	1.69 (1.62–1.76)
Kmer_8201	*nanA*	292 (83.7)	241 (55.5)	4.68 × 10^–17^	1.66 (1.55–1.76)

*OR, odds ratio.

## Discussion

**Figure 6:**
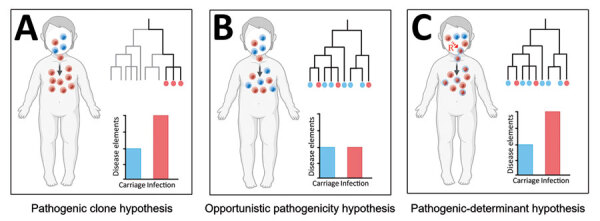
Pathogenicity models for genetically related disease-causing isolates used in study of disease-associated *Streptococcus pneumoniae* genetic variation. A) Pathogenic clone hypothesis; B) opportunistic pathogenicity hypothesis; C) pathogenic-determinant hypothesis.

To explore genomic differences between infection and carriage isolates, linking infection-associated genotypes with disease status is necessary. In our study, the most common serotypes for infection isolates (19F, 6B, 23F) were consistent with the results from other regions of China (*18*–*20*) but differed from those from the United States and Japan (*21*,*22*). Moreover, we observed considerable ST diversity among infection isolates; the most prevalent genotypes were ST271, ST320, and ST902, a finding consistent with those of previous studies in China but different from those in developed and developing countries (*23*–*25*). The resolution of MLST and serotyping for inferring isolate relatedness is limited, so we also used GPSCs to characterize and compare different lineages (*26*). The most prevalent GPSCs among the infection isolates were GPSC1, GPSC321, and GPSC852, which differed from those in the United States and South Africa (*27*). Our findings suggest that discrepancy in genotypes on a global scale may be associated with different pathogenicity and evolutionary directions. In our study, associations between specific genotypes (such as 19F and GPSC1) and disease status differed significantly, which is consistent with findings from a study in India (*28*). Our findings indicate that the presence of specific pathogenic clones may promote infection. In a simple pathogenicity model, all pathogenic clones would belong to specific clusters of genetically related disease-causing isolates (i.e., pathogenic clone hypothesis; Figure 6, panel A), which has been observed for *Staphylococcus aureus* and *S. pneumoniae* isolates (*29*,*30*). That pathogenicity model is not suitable for all *S. pneumoniae* clones because many infection isolates clustered in the same branches of phylogenetic tree as carriage isolates. In addition, traditional genotypes provide little power for identifying small genetic variations at the genomic level (*29*), suggesting that those genotypes only partially explain the pathogenicity of *S. pneumoniae*.

Using high-throughput genome sequencing technologies and bacterial GWAS methods to further explore high-dimensional genetic variation between infection and carriage isolates is essential, thereby revealing the pathogenicity-associated genetic elements of *S. pneumoniae.* According to the phylogenetic tree, we observed that infection isolates were markedly unevenly distributed across the phylogeny and also clustered with carriage isolates within several lineages, indicating that most lineages are capable of causing infection (i.e., opportunistic pathogenicity hypothesis; Figure 6, panel B). If this hypothesis is reasonable, then GWAS analyses would not detect numerous pathogenicity-associated k-mers. However, the LMM-based GWAS in our study detected 22,790 pathogenicity-associated k-mers. These findings suggest that the enrichment of genetic elements encoding pathogenicity traits may increase the pathogenicity of *S. pneumoniae* (i.e., pathogenic-determinant hypothesis; Figure 6, panel C), which is consistent with *Staphylococcus epidermidis* and avian pathogenic *Escherichia coli* (*30*,*31*). In this pathogenic-determinant model, horizontal gene transfer could spread genetic determinants in bacteria such as *S. pneumoniae* and *Klebsiella pneumoniae* (*32*–*34*), leading various clones to successfully cause disease.

High-throughput genomic data have brought substantial challenges to data analysis because of high-dimensional and highly correlated data structures. In our study, we identified infection-associated k-mers by using a 2-stage comprehensive GWAS analysis process, including LMM for initially screening pathogenic k-mers and multiple GWAS methods for further validation. In the final prediction model, we identified 8 k-mer predictors, which mapped to genes associated with adherence, immune regulation, antibiotic resistance, and exoenzyme. Of the adherence-related genes, *srtG1* and the LPxTG-type surface-anchored protein (*pitA*) are important components of the pneumococcal pilus-2, which plays a crucial role in promoting adhesion, colonization, and cellular invasion (*35*,*36*). Classification accuracy of the most important k-mer in *srtG1* was high by itself, and that of the additional validation RF analysis based on open datasets was similar, suggesting that this predictor has great potential for predicting pathogenic isolates in a clinical setting. Phosphorylcholine esterase (*cbpE*) plays an important role in modulating both the phosphorylcholine decoration of its surface and choline-bound surface adhesins, which may contribute to pneumococcal adherence and invasiveness (*37*). Capsular polysaccharide (CPS) is a major virulence factor in *S. pneumoniae*. Capsular polysaccharide protein C (CpsC) has been shown to affect the level of CPS expression and also regulate the assembly, export, and attachment of CPS to the cell wall (*38*). Pneumococcal surface protein A (PspA) plays role in preventing complement-mediated opsonization and is also capable of binding to lactoferrin, thereby preventing it from killing pneumococci (*39*). The infection-associated genes reported in our study (*cpsC* and *pspA*) are homologous to the genes associated with invasive pneumococci (*cpsA*, *cpsD*, and *pspC*) identified in previous studies (*11*,*12*), providing more evidence for *S. pneumoniae* pathogenicity. Neuraminidase A encoded by the *nanA* gene is an essential colonization factor for *S. pneumoniae* and promotes growth and survival of the bacteria in the upper respiratory tract (*40*). Antimicrobial drug use and abuse not only induce widespread multidrug-resistant pneumococci but also increase the susceptibility to invasive disease (*41*). For decades, penicillin has been the first choice for treatment of pneumococcal infection, and mutations in penicillin-binding proteins (PBPs) are essential for high-level penicillin resistance (*42*). Li et al. demonstrated that *pbp2b* and *pbp3* are associated with pneumococcal infection (*42*). One reason is that PBP2B and PBP3 are involved in the synthesis and growth of bacterial cell walls, which are crucial for the survival and virulence of pneumococci (*43*). In addition, a previous study revealed a potential association between penicillin resistance and GPSC1 (*44*), and our findings also showed that GPSC1 was associated with pneumococcal infection, suggesting that it cannot support a causal link between resistance and pneumococcal infection and may result from a lineage confounder. In summary, these infection-associated k-mers provide genetic evidence for revealing optimal risk factors for infection isolates, which may offer a theoretical basis for precise targeted interventions.

In this study, we attempted to use the comprehensive analysis strategy to identify pathogenic k-mers by well-characterized *S. pneumoniae* isolates from a single location so we could reduce redundancy of k-mer predictors, minimize false-positive associations, and avoid geographic variation. Our consensus findings of pathogenic k-mers from multiple GWAS methods may provide sufficient evidence for clarifying the complex multifactorial pathogenicity of *S. pneumoniae*. However, among the potential limitations, the first is that *S. pneumoniae* pathogenesis is a multifactorial and interacting process, but traditional GWAS methods identify the main effect of each genetic variation and ignore the complex gene-gene interactions (*45*). Therefore, future studies should use the enrichment theory to determine the core functions or pathways for risk genes, which may provide new insights for understanding pathogenesis at functional levels (*46*,*47*). Second, although k-mers can reflect variation in bacterial genomes, we mapped infection-associated k-mers in our study to reference genomes to identify pathogenic genes, which cannot cover complete genomic variation in the entire species. To overcome those issues, we developed the extended k-mer–based GWAS methods to detect phenotype-specific k-mers without relying on prior annotations or reference genomes (*48*,*49*). Third, our study focused mainly on noninvasive rather than invasive isolates, and *S. pneumoniae* can transition from carriage to infection, suggesting potential similarity in carriage and noninvasive infection isolates. To improve the statistical power and comparability of exploring disease-associated markers, we included infection isolates from children with confirmed associated symptoms and carriage isolates from asymptomatic healthy children.

In conclusion, our 2-stage GWAS analyses identified a subset of 8 pathogenic k-mers associated with adherence, antimicrobial resistance, and immune modulation, indicating that the enrichment of genetic elements encoding pathogenicity traits may increase the pathogenicity of *S. pneumoniae.* The best predictor for *S. pneumoniae* infection achieved a high classification accuracy, giving a very simple target for predicting pathogenic isolates in a clinical setting. These findings suggest the complex multifactorial nature of *S. pneumoniae* pathogenicity and provide genetic evidence for the evolution of virulence and development of precise targeted interventions.

**Appendix.** Additional information for study of disease-associated *Streptococcus pneumoniae* genetic variation.

## References

[1] Henriques-Normark B, Tuomanen EI. The pneumococcus: epidemiology, microbiology, and pathogenesis. Cold Spring Harb Perspect Med. 2013;3:a010215. 10.1101/cshperspect.a010215 23818515 PMC3685878

[2] Mancuso G, Midiri A, Gerace E, Biondo C. Bacterial antibiotic resistance: the most critical pathogens. Pathogens. 2021;10:1310. 10.3390/pathogens10101310 34684258 PMC8541462

[3] Subramanian K, Henriques-Normark B, Normark S. Emerging concepts in the pathogenesis of the *Streptococcus pneumoniae*: From nasopharyngeal colonizer to intracellular pathogen. Cell Microbiol. 2019;21:e13077. 10.1111/cmi.13077 31251447 PMC6899785

[4] Bogaert D, De Groot R, Hermans PW. Streptococcus *pneumoniae* colonisation: the key to pneumococcal disease. Lancet Infect Dis. 2004;4:144–54. 10.1016/S1473-3099(04)00938-7 14998500

[5] Yao KH, Yang YH. *Streptococcus pneumoniae* diseases in Chinese children: past, present and future. Vaccine. 2008;26:4425–33. 10.1016/j.vaccine.2008.06.052 18602435

[6] Kadioglu A, Weiser JN, Paton JC, Andrew PW. The role of *Streptococcus pneumoniae* virulence factors in host respiratory colonization and disease. Nat Rev Microbiol. 2008;6:288–301. 10.1038/nrmicro1871 18340341

[7] Piet JR, Geldhoff M, van Schaik BD, Brouwer MC, Valls Seron M, Jakobs ME, et al. *Streptococcus pneumoniae* arginine synthesis genes promote growth and virulence in pneumococcal meningitis. J Infect Dis. 2014;209:1781–91. 10.1093/infdis/jit818 24338350

[8] Tunjungputri RN, Mobegi FM, Cremers AJ, van der Gaast-de Jongh CE, Ferwerda G, Meis JF, et al. Phage-derived protein induces increased platelet activation and is associated with mortality in patients with invasive pneumococcal disease. MBio. 2017;8:e01984–16. 10.1128/mBio.01984-16 28096486 PMC5241397

[9] Chaguza C, Ebruke C, Senghore M, Lo SW, Tientcheu PE, Gladstone RA, et al. Comparative genomics of disease and carriage serotype 1 pneumococci. Genome Biol Evol. 2022;14:evac052. 10.1093/gbe/evac052 PMC9048925 35439297

[10] The CRyPTIC Consortium. Genome-wide association studies of global *Mycobacterium tuberculosis* resistance to 13 antimicrobials in 10,228 genomes identify new resistance mechanisms. PLoS Biol. 2022;20:e3001755. 10.1371/journal.pbio.3001755 35944070 PMC9363015

[11] Lees JA, Ferwerda B, Kremer PHC, Wheeler NE, Serón MV, Croucher NJ, et al. Joint sequencing of human and pathogen genomes reveals the genetics of pneumococcal meningitis. Nat Commun. 2019;10:2176. 10.1038/s41467-019-09976-3 31092817 PMC6520353

[12] Obolski U, Gori A, Lourenço J, Thompson C, Thompson R, French N, et al. Identifying genes associated with invasive disease in *S. pneumoniae* by applying a machine learning approach to whole genome sequence typing data. Sci Rep. 2019;9:4049. 10.1038/s41598-019-40346-7 30858412 PMC6411942

[13] Lees JA, Vehkala M, Välimäki N, Harris SR, Chewapreecha C, Croucher NJ, et al. Sequence element enrichment analysis to determine the genetic basis of bacterial phenotypes. Nat Commun. 2016;7:12797. 10.1038/ncomms12797 27633831 PMC5028413

[14] Gupta PK. Quantitative genetics: pan-genomes, SVs, and k-mers for GWAS. Trends Genet. 2021;37:868–71. 10.1016/j.tig.2021.05.006 34183185

[15] Lees JA, Galardini M, Bentley SD, Weiser JN, Corander J. pyseer: a comprehensive tool for microbial pangenome-wide association studies. Bioinformatics. 2018;34:4310–2. 10.1093/bioinformatics/bty539 30535304 PMC6289128

[16] Pedregosa F, Varoquaux G, Gramfort A, Michel V, Thirion B, Grisel O, et al. Scikit-learn: machine learning in Python. J Mach Learn Res. 2011;12:2825–30. 10.48550/arXiv.1201.0490

[17] Genuer R, Poggi J-M, Tuleau-Malot C. VSURF: an R package for variable selection using random forests. R J. 2015;7:19–33. 10.32614/RJ-2015-018

[18] Geng Q, Zhang T, Ding Y, Tao Y, Lin Y, Wang Y, et al. Molecular characterization and antimicrobial susceptibility of *Streptococcus pneumoniae* isolated from children hospitalized with respiratory infections in Suzhou, China. PLoS One. 2014;9:e93752. 10.1371/journal.pone.0093752 24710108 PMC3977860

[19] Shi W, Li J, Dong F, Qian S, Liu G, Xu B, et al. Serotype distribution, antibiotic resistance pattern, and multilocus sequence types of invasive *Streptococcus pneumoniae* isolates in two tertiary pediatric hospitals in Beijing prior to PCV13 availability. Expert Rev Vaccines. 2019;18:89–94. 10.1080/14760584.2019.1557523 30526145

[20] Yu YY, Xie XH, Ren L, Deng Y, Gao Y, Zhang Y, et al. Epidemiological characteristics of nasopharyngeal *Streptococcus pneumoniae* strains among children with pneumonia in Chongqing, China. Sci Rep. 2019;9:3324. 10.1038/s41598-019-40088-6 30824811 PMC6397308

[21] Suaya JA, Mendes RE, Sings HL, Arguedas A, Reinert RR, Jodar L, et al. *Streptococcus pneumoniae* serotype distribution and antimicrobial nonsusceptibility trends among adults with pneumonia in the United States, 2009‒2017. J Infect. 2020;81:557–66. 10.1016/j.jinf.2020.07.035 32739491

[22] Yanagihara K, Kosai K, Mikamo H, Mukae H, Takesue Y, Abe M, et al. Serotype distribution and antimicrobial susceptibility of *Streptococcus pneumoniae* associated with invasive pneumococcal disease among adults in Japan. Int J Infect Dis. 2021;102:260–8. 10.1016/j.ijid.2020.10.017 33065297

[23] Yan Z, Cui Y, Huang X, Lei S, Zhou W, Tong W, et al. Molecular characterization based on whole-genome sequencing of *Streptococcus pneumoniae* in children living in southwest China during 2017–2019. Front Cell Infect Microbiol. 2021;11:726740. 10.3389/fcimb.2021.726740 34796125 PMC8593041

[24] Kellner JD, Ricketson LJ, Demczuk WHB, Martin I, Tyrrell GJ, Vanderkooi OG, et al. Whole-genome analysis of *Streptococcus pneumoniae* serotype 4 causing outbreak of invasive pneumococcal disease, Alberta, Canada. Emerg Infect Dis. 2021;27:1867–75. 10.3201/eid2707.204403 34152965 PMC8237880

[25] Vorobieva S Jensen V, Furberg AS, Slotved HC, Bazhukova T, Haldorsen B, Caugant DA, et al. Epidemiological and molecular characterization of *Streptococcus pneumoniae* carriage strains in pre-school children in Arkhangelsk, northern European Russia, prior to the introduction of conjugate pneumococcal vaccines. BMC Infect Dis. 2020;20:279. 10.1186/s12879-020-04998-5 32293324 PMC7161136

[26] Gladstone RA, Lo SW, Lees JA, Croucher NJ, van Tonder AJ, Corander J, et al.; Global Pneumococcal Sequencing Consortium. International genomic definition of pneumococcal lineages, to contextualise disease, antibiotic resistance and vaccine impact. EBioMedicine. 2019;43:338–46. 10.1016/j.ebiom.2019.04.021 31003929 PMC6557916

[27] Lo SW, Gladstone RA, van Tonder AJ, Lees JA, du Plessis M, Benisty R, et al.; Global Pneumococcal Sequencing Consortium. Pneumococcal lineages associated with serotype replacement and antibiotic resistance in childhood invasive pneumococcal disease in the post-PCV13 era: an international whole-genome sequencing study. Lancet Infect Dis. 2019;19:759–69. 10.1016/S1473-3099(19)30297-X 31196809 PMC7641901

[28] Nagaraj G, Govindan V, Ganaie F, Venkatesha VT, Hawkins PA, Gladstone RA, et al. *Streptococcus pneumoniae* genomic datasets from an Indian population describing pre-vaccine evolutionary epidemiology using a whole genome sequencing approach. Microb Genom. 2021;7:000645. 10.1099/mgen.0.000645 34494953 PMC8715438

[29] Harris SR, Feil EJ, Holden MT, Quail MA, Nickerson EK, Chantratita N, et al. Evolution of MRSA during hospital transmission and intercontinental spread. Science. 2010;327:469–74. 10.1126/science.1182395 20093474 PMC2821690

[30] Méric G, Mageiros L, Pensar J, Laabei M, Yahara K, Pascoe B, et al. Disease-associated genotypes of the commensal skin bacterium *Staphylococcus epidermidis.* Nat Commun. 2018;9:5034. 10.1038/s41467-018-07368-7 30487573 PMC6261936

[31] Mageiros L, Méric G, Bayliss SC, Pensar J, Pascoe B, Mourkas E, et al. Genome evolution and the emergence of pathogenicity in avian *Escherichia coli.* Nat Commun. 2021;12:765. 10.1038/s41467-021-20988-w 33536414 PMC7858641

[32] Arnold BJ, Huang IT, Hanage WP. Horizontal gene transfer and adaptive evolution in bacteria. Nat Rev Microbiol. 2022;20:206–18. 10.1038/s41579-021-00650-4 34773098

[33] Wyres KL, Wick RR, Judd LM, Froumine R, Tokolyi A, Gorrie CL, et al. Distinct evolutionary dynamics of horizontal gene transfer in drug resistant and virulent clones of *Klebsiella pneumoniae.* PLoS Genet. 2019;15:e1008114. 10.1371/journal.pgen.1008114 30986243 PMC6483277

[34] Salvadori G, Junges R, Morrison DA, Petersen FC. Competence in *Streptococcus pneumoniae* and close commensal relatives: mechanisms and implications. Front Cell Infect Microbiol. 2019;9:94. 10.3389/fcimb.2019.00094 31001492 PMC6456647

[35] Bagnoli F, Moschioni M, Donati C, Dimitrovska V, Ferlenghi I, Facciotti C, et al. A second pilus type in *Streptococcus pneumoniae* is prevalent in emerging serotypes and mediates adhesion to host cells. J Bacteriol. 2008;190:5480–92. 10.1128/JB.00384-08 18515415 PMC2493256

[36] Dzaraly ND, Muthanna A, Mohd Desa MN, Taib NM, Masri SN, Rahman NIA, et al. Pilus islets and the clonal spread of piliated *Streptococcus pneumoniae*: A review. Int J Med Microbiol. 2020;310:151449. 10.1016/j.ijmm.2020.151449 33092697

[37] Hermoso JA, Lagartera L, González A, Stelter M, García P, Martínez-Ripoll M, et al. Insights into pneumococcal pathogenesis from the crystal structure of the modular teichoic acid phosphorylcholine esterase Pce. Nat Struct Mol Biol. 2005;12:533–8. 10.1038/nsmb940 15895092

[38] Kadioglu A, Weiser JN, Paton JC, Andrew PW. The role of *Streptococcus pneumoniae* virulence factors in host respiratory colonization and disease. Nat Rev Microbiol. 2008;6:288–301. 10.1038/nrmicro1871 18340341

[39] Marquart ME. Pathogenicity and virulence of *Streptococcus pneumoniae*: Cutting to the chase on proteases. Virulence. 2021;12:766–87. 10.1080/21505594.2021.1889812 33660565 PMC7939560

[40] Brittan JL, Buckeridge TJ, Finn A, Kadioglu A, Jenkinson HF. Pneumococcal neuraminidase A: an essential upper airway colonization factor for *Streptococcus pneumoniae.* Mol Oral Microbiol. 2012;27:270–83. 10.1111/j.2041-1014.2012.00658.x 22759312

[41] Navarro-Torné A, Dias JG, Hruba F, Lopalco PL, Pastore-Celentano L, Gauci AJ; Invasive Pneumococcal Disease Study Group. Risk factors for death from invasive pneumococcal disease, Europe, 2010. Emerg Infect Dis. 2015;21:417–25. 10.3201/eid2103.140634 25693604 PMC4344260

[42] Li Y, Metcalf BJ, Chochua S, Li Z, Gertz RE Jr, Walker H, et al. Penicillin-binding protein transpeptidase signatures for tracking and predicting β-lactam resistance levels in *Streptococcus pneumoniae.* MBio. 2016;7:e00756–16. 10.1128/mBio.00756-16 27302760 PMC4916381

[43] Gibson PS, Veening JW. Gaps in the wall: understanding cell wall biology to tackle amoxicillin resistance in *Streptococcus pneumoniae.* Curr Opin Microbiol. 2023;72:102261. 10.1016/j.mib.2022.102261 36638546

[44] Egorova E, Kumar N, Gladstone RA, Urban Y, Voropaeva E, Chaplin AV, et al. Key features of pneumococcal isolates recovered in Central and Northwestern Russia in 2011-2018 determined through whole-genome sequencing. Microb Genom. 2022;8:mgen000851. 10.1099/mgen.0.000851 36112007 PMC9676041

[45] Bai G, Vidal JE. Editorial: molecular pathogenesis of *Pneumococcus.* Front Cell Infect Microbiol. 2017;7:310. 10.3389/fcimb.2017.00310 28744450 PMC5504239

[46] Chen L, Zhang YH, Wang S, Zhang Y, Huang T, Cai YD. Prediction and analysis of essential genes using the enrichments of gene ontology and KEGG pathways. PLoS One. 2017;12:e0184129. 10.1371/journal.pone.0184129 28873455 PMC5584762

[47] Kanehisa M, Furumichi M, Sato Y, Kawashima M, Ishiguro-Watanabe M. KEGG for taxonomy-based analysis of pathways and genomes. Nucleic Acids Res. 2023;51(D1):D587–92. 10.1093/nar/gkac963 36300620 PMC9825424

[48] Jaillard M, Lima L, Tournoud M, Mahé P, van Belkum A, Lacroix V, et al. A fast and agnostic method for bacterial genome-wide association studies: Bridging the gap between k-mers and genetic events. PLoS Genet. 2018;14:e1007758. 10.1371/journal.pgen.1007758 30419019 PMC6258240

[49] Aun E, Brauer A, Kisand V, Tenson T, Remm M. A k-mer-based method for the identification of phenotype-associated genomic biomarkers and predicting phenotypes of sequenced bacteria. PLOS Comput Biol. 2018;14:e1006434. 10.1371/journal.pcbi.1006434 30346947 PMC6211763

