# Supplementary material for: Disease-Associated Streptococcus pneumoniae Genetic Variation
Source: Emerg Infect Dis. 2024 Jan;30(1):39–49. doi: 10.3201/eid3001.221927 (PMC10756394; doi:10.3201/eid3001.221927)
Supplement: Supplementary file 1 — Appendix. Additional information for study of disease-associated Streptococcus pneumoniae genetic variation. [file 22-1927-Techapp-s1.pdf]

Article DOI: https://doi.org/10.3201/eid3001.221927

EID cannot ensure accessibility for supplementary materials supplied by authors. Readers who have difficulty accessing supplementary content should contact the authors for assistance.

Disease-Associated *Streptococcus pneumoniae* Genetic Variation

Appendix

Appendix Table 1. Detailed information of 783 Streptococcus pneumoniae isolates in our study

Bioproject accession	SRA accession number	Disease phenotypes	Isolation source	Sex	Age (years)	GPSCs	Serotypes	MLST
PRJNA976286	SRR24861694	Carriage	NP swab	M	3	GPSC4	14	ST876
PRJNA976286	SRR24861693	Carriage	NP swab	M	3	GPSC5	23A	ST338
PRJNA976286	SRR24861614	Carriage	NP swab	M	4	GPSC4	14	ST876
PRJNA976286	SRR24861040	Carriage	NP swab	F	3	GPSC321	6B	ST902
PRJNA976286	SRR24861025	Carriage	NP swab	F	3	GPSC4	14	ST876
PRJNA976286	SRR24861345	Carriage	NP swab	F	3	GPSC852	6A	-
PRJNA976286	SRR24861330	Carriage	NP swab	M	3	GPSC1	19F	-
PRJNA976286	SRR24861091	Carriage	NP swab	M	3	GPSC321	6B	ST902
PRJNA976286	SRR24861445	Carriage	NP swab	M	2	GPSC1	19F	ST271
PRJNA976286	SRR24861082	Carriage	NP swab	F	3	GPSC73	11A	ST99
PRJNA976286	SRR24861692	Carriage	NP swab	M	6	GPSC321	6B	ST902
PRJNA976286	SRR24861681	Carriage	NP swab	F	3	GPSC69	15A	ST11972
PRJNA976286	SRR24861670	Carriage	NP swab	M	5	GPSC10	23A	ST9396
PRJNA976286	SRR24861659	Carriage	NP swab	M	6	GPSC45	34	-
PRJNA976286	SRR24861648	Carriage	NP swab	F	5	GPSC321	6B	ST902
PRJNA976286	SRR24861637	Carriage	NP swab	M	6	GPSC1	19F	ST320
PRJNA976286	SRR24861274	Carriage	NP swab	F	5	GPSC4	14	ST876
PRJNA976286	SRR24861263	Carriage	NP swab	F	3	GPSC4	14	ST876
PRJNA976286	SRR24861252	Carriage	NP swab	M	3	GPSC45	34	ST9395
PRJNA976286	SRR24861625	Carriage	NP swab	M	3	GPSC1	19A	ST320
PRJNA976286	SRR24861613	Carriage	NP swab	F	3	GPSC1	19F	ST271
PRJNA976286	SRR24861602	Carriage	NP swab	F	4	GPSC45	34	ST9395
PRJNA976286	SRR24861239	Carriage	NP swab	M	4	GPSC321	6B	ST902
PRJNA976286	SRR24861228	Carriage	NP swab	M	4	GPSC321	6B	ST902
PRJNA976286	SRR24861217	Carriage	NP swab	M	4	GPSC1	19A	ST320

Bioproject accession	SRA accession number	Disease phenotypes	Isolation source	Sex	Age (years)	GPSCs	Serotypes	MLST
PRJNA976286	SRR24861430	Carriage	NP swab	F	3	GPSC69	15A	ST6011
PRJNA976286	SRR24861419	Carriage	NP swab	F	3	GPSC321	6B	ST902
PRJNA976286	SRR24861408	Carriage	NP swab	F	3	GPSC14	23F	ST242
PRJNA976286	SRR24861062	Carriage	NP swab	M	5	GPSC904;9	15A	ST63
PRJNA976286	SRR24861051	Carriage	NP swab	M	5	GPSC1	19A	ST320
PRJNA976286	SRR24861598	Carriage	NP swab	M	4	GPSC904;9	15A	ST63
PRJNA976286	SRR24861587	Carriage	NP swab	M	5	GPSC321	6B	ST902
PRJNA976286	SRR24861576	Carriage	NP swab	M	4	GPSC321	6B	ST902
PRJNA976286	SRR24861213	Carriage	NP swab	F	4	GPSC904;9	15A	ST63
PRJNA976286	SRR24861202	Carriage	NP swab	M	5	GPSC1	19A	ST320
PRJNA976286	SRR24861191	Carriage	NP swab	F	5	GPSC904;9	15A	ST63
PRJNA976286	SRR24861404	Carriage	NP swab	F	5	GPSC904;9	15A	ST63
PRJNA976286	SRR24861393	Carriage	NP swab	F	4	GPSC904;9	15A	ST63
PRJNA976286	SRR24861382	Carriage	NP swab	F	5	GPSC152	15B	ST7768
PRJNA976286	SRR24861036	Carriage	NP swab	M	5	GPSC152	15B	ST3397
PRJNA976286	SRR24861024	Carriage	NP swab	M	5	GPSC321	6B	ST902
PRJNA976286	SRR24861796	Carriage	NP swab	F	5	GPSC1	19A	ST320
PRJNA976286	SRR24861561	Carriage	NP swab	M	6	GPSC45	34	ST11964
PRJNA976286	SRR24861550	Carriage	NP swab	M	6	GPSC321	6B	ST902
PRJNA976286	SRR24861539	Carriage	NP swab	M	6	GPSC904;9	15A	ST63
PRJNA976286	SRR24861176	Carriage	NP swab	M	6	GPSC321	6B	ST902
PRJNA976286	SRR24861165	Carriage	NP swab	F	5	GPSC321	6B	ST902
PRJNA976286	SRR24861154	Carriage	NP swab	F	5	GPSC1	19F	ST271
PRJNA976286	SRR24861367	Carriage	NP swab	F	6	GPSC45	34	-
PRJNA976286	SRR24861356	Carriage	NP swab	M	6	-	-	-
PRJNA976286	SRR24861344	Carriage	NP swab	M	5	-	-	ST10236
PRJNA976286	SRR24861781	Carriage	NP swab	M	6	GPSC321	6B	ST902
PRJNA976286	SRR24861770	Carriage	NP swab	F	4	GPSC321	6B	ST902
PRJNA976286	SRR24861759	Carriage	NP swab	F	5	GPSC321	6B	ST902
PRJNA976286	SRR24861524	Carriage	NP swab	M	5	GPSC1	19A	ST320

Bioproject accession	SRA accession number	Disease phenotypes	Isolation source	Sex	Age (years)	GPSCs	Serotypes	MLST
PRJNA976286	SRR24861513	Carriage	NP swab	M	5	GPSC1	19A	ST320
PRJNA976286	SRR24861150	Carriage	NP swab	M	4	GPSC321	6B	ST902
PRJNA976286	SRR24861139	Carriage	NP swab	F	5	GPSC321	6B	ST902
PRJNA976286	SRR24861128	Carriage	NP swab	F	5	GPSC321	6B	ST902
PRJNA976286	SRR24861341	Carriage	NP swab	M	3	GPSC4	14	ST876
PRJNA976286	SRR24861329	Carriage	NP swab	M	2	GPSC45	34	ST9395
PRJNA976286	SRR24861318	Carriage	NP swab	M	4	GPSC45	34	ST9395
PRJNA976286	SRR24861755	Carriage	NP swab	F	3	GPSC23	6B	ST8526
PRJNA976286	SRR24861744	Carriage	NP swab	M	3	GPSC23	6B	ST8526
PRJNA976286	SRR24861733	Carriage	NP swab	F	4	GPSC45	34	ST9395
PRJNA976286	SRR24861498	Carriage	NP swab	M	4	GPSC45	34	ST9395
PRJNA976286	SRR24861487	Carriage	NP swab	M	4	GPSC45	34	-
PRJNA976286	SRR24861476	Carriage	NP swab	M	4	GPSC45	34	ST9395
PRJNA976286	SRR24861113	Carriage	NP swab	M	3	GPSC69	15A	ST6011
PRJNA976286	SRR24861102	Carriage	NP swab	M	5	-	-	ST7401
PRJNA976286	SRR24861090	Carriage	NP swab	M	6	GPSC321	6B	ST902
PRJNA976286	SRR24861303	Carriage	NP swab	M	6	-	16F	ST6542
PRJNA976286	SRR24861292	Carriage	NP swab	M	7	-	-	ST10236
PRJNA976286	SRR24861281	Carriage	NP swab	M	6	GPSC321	6B	ST902
PRJNA976286	SRR24861718	Carriage	NP swab	F	5	GPSC321	6B	ST902
PRJNA976286	SRR24861707	Carriage	NP swab	F	5	GPSC69	15A	-
PRJNA976286	SRR24861696	Carriage	NP swab	F	4	GPSC4	14	ST876
PRJNA976286	SRR24861461	Carriage	NP swab	F	5	GPSC69	15A	-
PRJNA976286	SRR24861450	Carriage	NP swab	M	4	GPSC4	14	ST876
PRJNA976286	SRR24861446	Carriage	NP swab	M	5	GPSC69	15A	-
PRJNA976286	SRR24861444	Carriage	NP swab	F	6	GPSC69	15A	ST11972
PRJNA976286	SRR24861443	Carriage	NP swab	F	6	GPSC158	16F	ST12671
PRJNA976286	SRR24861442	Carriage	NP swab	M	6	GPSC69	15A	ST11972
PRJNA976286	SRR24861441	Carriage	NP swab	M	6	GPSC230	35C	ST7752
PRJNA976286	SRR24861440	Carriage	NP swab	M	6	GPSC230	35C	ST7752
PRJNA976286	SRR24861439	Carriage	NP swab	M	6	GPSC230	35C	ST7752
PRJNA976286	SRR24861086	Carriage	NP swab	F	5	GPSC1	19F	ST271
PRJNA976286	SRR24861085	Carriage	NP swab	F	5	GPSC321	6B	ST902
PRJNA976286	SRR24861084	Carriage	NP swab	F	5	GPSC152	15C	ST6555
PRJNA976286	SRR24861083	Carriage	NP swab	F	6	GPSC230	13	ST2754
PRJNA976286	SRR24861081	Carriage	NP swab	F	6	GPSC230	13	ST2754
PRJNA976286	SRR24861080	Carriage	NP swab	M	5	-	6B	-

Bioproject accession	SRA accession number	Disease phenotypes	Isolation source	Sex	Age (years)	GPSCs	Serotypes	MLST
PRJNA976286	SRR24861079	Carriage	NP swab	M	6	GPSC152	15C	ST6555
PRJNA976286	SRR24861078	Carriage	NP swab	M	6	GPSC23	6B	ST90
PRJNA976286	SRR24861077	Carriage	NP swab	M	5	GPSC165	34	-
PRJNA976286	SRR24861076	Carriage	NP swab	F	2	GPSC69	15A	ST11972
PRJNA976286	SRR24861075	Carriage	NP swab	M	4	GPSC244	6C	-
PRJNA976286	SRR24861074	Carriage	NP swab	F	3	GPSC244	6C	-
PRJNA976286	SRR24861073	Carriage	NP swab	M	4	GPSC45	34	-
PRJNA976286	SRR24861072	Carriage	NP swab	F	3	GPSC244	6C	-
PRJNA976286	SRR24861691	Carriage	NP swab	M	3	GPSC244	6C	-
PRJNA976286	SRR24861690	Carriage	NP swab	F	4	GPSC158	16F	-
PRJNA976286	SRR24861689	Carriage	NP swab	F	3	GPSC158	16F	-
PRJNA976286	SRR24861688	Carriage	NP swab	F	4	GPSC23	6B	ST90
PRJNA976286	SRR24861687	Carriage	NP swab	M	3	-	6B	-
PRJNA976286	SRR24861686	Carriage	NP swab	F	4	GPSC244	6C	-
PRJNA976286	SRR24861685	Carriage	NP swab	M	4	GPSC69	15A	-
PRJNA976286	SRR24861684	Carriage	NP swab	M	4	GPSC158	16F	-
PRJNA976286	SRR24861683	Carriage	NP swab	M	3	GPSC158	16F	-
PRJNA976286	SRR24861682	Carriage	NP swab	M	4	GPSC244	6C	-
PRJNA976286	SRR24861680	Carriage	NP swab	F	3	GPSC45	34	-
PRJNA976286	SRR24861679	Carriage	NP swab	F	4	GPSC69	15A	ST11972
PRJNA976286	SRR24861678	Carriage	NP swab	M	4	GPSC69	15A	ST11972
PRJNA976286	SRR24861677	Carriage	NP swab	F	4	GPSC69	15A	ST11972
PRJNA976286	SRR24861676	Carriage	NP swab	M	4	GPSC45	6A	-
PRJNA976286	SRR24861675	Carriage	NP swab	F	4	-	14	-
PRJNA976286	SRR24861674	Carriage	NP swab	M	4	GPSC69	15A	ST11972
PRJNA976286	SRR24861673	Carriage	NP swab	M	4	GPSC69	15A	ST11972
PRJNA976286	SRR24861672	Carriage	NP swab	M	4	GPSC852	6B	ST3173
PRJNA976286	SRR24861671	Carriage	NP swab	M	3	GPSC165	34	-
PRJNA976286	SRR24861669	Carriage	NP swab	M	4	GPSC69	15A	ST11972
PRJNA976286	SRR24861668	Carriage	NP swab	M	4	GPSC23	6B	ST90
PRJNA976286	SRR24861667	Carriage	NP swab	F	4	GPSC23	6B	ST90
PRJNA976286	SRR24861666	Carriage	NP swab	F	4	GPSC23	6B	ST90
PRJNA976286	SRR24861665	Carriage	NP swab	M	3	GPSC23	6B	ST90
PRJNA976286	SRR24861664	Carriage	NP swab	M	4	GPSC4	14	ST876
PRJNA976286	SRR24861663	Carriage	NP swab	M	4	GPSC186	35B	ST6327
PRJNA976286	SRR24861662	Carriage	NP swab	M	4	GPSC69	15A	ST11972
PRJNA976286	SRR24861661	Carriage	NP swab	M	4	GPSC23	6B	ST90

Bioproject accession	SRA accession number	Disease phenotypes	Isolation source	Sex	Age (years)	GPSCs	Serotypes	MLST
PRJNA976286	SRR24861660	Carriage	NP swab	M	4	GPSC45	6A	-
PRJNA976286	SRR24861658	Carriage	NP swab	M	3	GPSC186	35B	ST6327
PRJNA976286	SRR24861657	Carriage	NP swab	F	2	GPSC69	15A	ST11972
PRJNA976286	SRR24861656	Carriage	NP swab	F	6	GPSC212	35C	ST5972
PRJNA976286	SRR24861655	Carriage	NP swab	M	6	GPSC1	19A	ST320
PRJNA976286	SRR24861654	Carriage	NP swab	F	4	GPSC1	19F	ST271
PRJNA976286	SRR24861653	Carriage	NP swab	F	4	GPSC23	6B	ST90
PRJNA976286	SRR24861652	Carriage	NP swab	M	5	GPSC23	6B	ST90
PRJNA976286	SRR24861651	Carriage	NP swab	M	4	GPSC1	19F	ST271
PRJNA976286	SRR24861650	Carriage	NP swab	M	4	GPSC23	6B	ST90
PRJNA976286	SRR24861649	Carriage	NP swab	F	4	GPSC1	19F	ST236
PRJNA976286	SRR24861647	Carriage	NP swab	M	3	GPSC1	19F	ST236
PRJNA976286	SRR24861646	Carriage	NP swab	M	4	-	19A	ST10236
PRJNA976286	SRR24861645	Carriage	NP swab	F	4	GPSC23	6B	ST90
PRJNA976286	SRR24861644	Carriage	NP swab	M	3	GPSC152	15C	ST6555
PRJNA976286	SRR24861643	Carriage	NP swab	M	4	GPSC23	6B	ST90
PRJNA976286	SRR24861642	Carriage	NP swab	M	3	GPSC45	34	-
PRJNA976286	SRR24861641	Carriage	NP swab	M	4	GPSC152	15C	ST6555
PRJNA976286	SRR24861640	Carriage	NP swab	F	3	GPSC23	6B	ST90
PRJNA976286	SRR24861639	Carriage	NP swab	M	3	GPSC23	6B	ST90
PRJNA976286	SRR24861638	Carriage	NP swab	M	6	GPSC45	34	-
PRJNA976286	SRR24861636	Carriage	NP swab	M	5	GPSC45	34	-
PRJNA976286	SRR24861635	Carriage	NP swab	M	6	GPSC45	34	-
PRJNA976286	SRR24861634	Carriage	NP swab	F	5	GPSC23	6B	ST90
PRJNA976286	SRR24861633	Carriage	NP swab	F	6	-	6B	-
PRJNA976286	SRR24861632	Carriage	NP swab	M	5	GPSC23	6B	ST90
PRJNA976286	SRR24861631	Carriage	NP swab	M	5	GPSC177	35F	-
PRJNA976286	SRR24861278	Carriage	NP swab	M	4	-	-	ST10236
PRJNA976286	SRR24861277	Carriage	NP swab	M	5	GPSC152	15C	ST6555
PRJNA976286	SRR24861276	Carriage	NP swab	M	5	GPSC23	6B	ST90
PRJNA976286	SRR24861275	Carriage	NP swab	M	5	GPSC23	6B	ST90
PRJNA976286	SRR24861273	Carriage	NP swab	M	5	GPSC23	6B	ST90
PRJNA976286	SRR24861272	Carriage	NP swab	M	5	GPSC321	6B	ST902
PRJNA976286	SRR24861271	Carriage	NP swab	M	5	GPSC23	6B	-
PRJNA976286	SRR24861270	Carriage	NP swab	M	5	GPSC4	14	ST876
PRJNA976286	SRR24861269	Carriage	NP swab	M	5	GPSC152	15C	ST6555
PRJNA976286	SRR24861268	Carriage	NP swab	F	4	-	-	ST10236

Bioproject accession	SRA accession number	Disease phenotypes	Isolation source	Sex	Age (years)	GPSCs	Serotypes	MLST
PRJNA976286	SRR24861267	Carriage	NP swab	F	3	GPSC177	35F	-
PRJNA976286	SRR24861266	Carriage	NP swab	F	4	-	6B	ST10236
PRJNA976286	SRR24861265	Carriage	NP swab	F	4	GPSC1	19F	ST236
PRJNA976286	SRR24861264	Carriage	NP swab	M	4	GPSC152	15B	ST6555
PRJNA976286	SRR24861262	Carriage	NP swab	F	4	GPSC23	6B	ST90
PRJNA976286	SRR24861261	Carriage	NP swab	M	4	GPSC165	34	ST7753
PRJNA976286	SRR24861260	Carriage	NP swab	F	2	GPSC10	23A	ST6227
PRJNA976286	SRR24861259	Carriage	NP swab	M	2	GPSC23	6B	ST90
PRJNA976286	SRR24861258	Carriage	NP swab	M	2	GPSC23	6B	ST90
PRJNA976286	SRR24861257	Carriage	NP swab	F	6	GPSC12	3	ST505
PRJNA976286	SRR24861256	Carriage	NP swab	M	4	GPSC47	6B	ST386
PRJNA976286	SRR24861255	Carriage	NP swab	M	5	-	-	-
PRJNA976286	SRR24861254	Carriage	NP swab	F	5	GPSC45	34	-
PRJNA976286	SRR24861253	Carriage	NP swab	M	5	GPSC152	15C	ST6555
PRJNA976286	SRR24861251	Carriage	NP swab	M	5	GPSC1	19F	ST271
PRJNA976286	SRR24861250	Carriage	NP swab	M	5	GPSC152	15C	ST6555
PRJNA976286	SRR24861249	Carriage	NP swab	F	4	GPSC152	15C	ST6555
PRJNA976286	SRR24861248	Carriage	NP swab	M	3	GPSC321	6B	ST902
PRJNA976286	SRR24861247	Carriage	NP swab	F	3	GPSC321	6B	ST902
PRJNA976286	SRR24861630	Carriage	NP swab	M	3	GPSC212	35A	ST7751
PRJNA976286	SRR24861629	Carriage	NP swab	M	3	GPSC45	34	-
PRJNA976286	SRR24861628	Carriage	NP swab	M	2	GPSC321	6B	ST902
PRJNA976286	SRR24861627	Carriage	NP swab	F	3	GPSC321	6B	ST902
PRJNA976286	SRR24861626	Carriage	NP swab	F	3	GPSC212	35A	ST7751
PRJNA976286	SRR24861624	Carriage	NP swab	F	3	GPSC321	6B	ST902
PRJNA976286	SRR24861623	Carriage	NP swab	M	3	GPSC321	6B	ST902
PRJNA976286	SRR24861622	Carriage	NP swab	M	3	GPSC321	6B	ST902
PRJNA976286	SRR24861621	Carriage	NP swab	F	3	GPSC12	3	ST180
PRJNA976286	SRR24861620	Carriage	NP swab	F	3	GPSC10	23A	ST9396
PRJNA976286	SRR24861619	Carriage	NP swab	F	4	GPSC212	35A	ST7751
PRJNA976286	SRR24861618	Carriage	NP swab	F	4	-	6B	ST63
PRJNA976286	SRR24861617	Carriage	NP swab	F	4	GPSC10	23A	ST9396
PRJNA976286	SRR24861616	Carriage	NP swab	M	3	GPSC10	23A	ST9396
PRJNA976286	SRR24861615	Carriage	NP swab	F	4	GPSC10	23A	ST9396
PRJNA976286	SRR24861612	Carriage	NP swab	F	4	GPSC10	23A	ST9396
PRJNA976286	SRR24861611	Carriage	NP swab	M	3	GPSC10	23A	ST9396
PRJNA976286	SRR24861610	Carriage	NP swab	M	4	GPSC10	23A	ST9396

Bioproject accession	SRA accession number	Disease phenotypes	Isolation source	Sex	Age (years)	GPSCs	Serotypes	MLST
PRJNA976286	SRR24861609	Carriage	NP swab	F	4	GPSC321	6B	ST902
PRJNA976286	SRR24861608	Carriage	NP swab	M	4	GPSC10	23A	ST9396
PRJNA976286	SRR24861607	Carriage	NP swab	M	4	GPSC10	23A	ST9396
PRJNA976286	SRR24861606	Carriage	NP swab	F	4	GPSC158	16F	ST8250
PRJNA976286	SRR24861605	Carriage	NP swab	F	4	GPSC1	19F	ST271
PRJNA976286	SRR24861604	Carriage	NP swab	M	4	GPSC321	6B	ST902
PRJNA976286	SRR24861603	Carriage	NP swab	M	3	GPSC10	23A	ST9396
PRJNA976286	SRR24861601	Carriage	NP swab	F	3	GPSC10	23A	-
PRJNA976286	SRR24861600	Carriage	NP swab	F	4	GPSC10	23A	ST9396
PRJNA976286	SRR24861599	Carriage	NP swab	F	4	GPSC10	23A	ST9396
PRJNA976286	SRR24861246	Carriage	NP swab	F	4	GPSC10	23A	ST9396
PRJNA976286	SRR24861245	Carriage	NP swab	F	4	GPSC10	23A	-
PRJNA976286	SRR24861244	Carriage	NP swab	F	4	GPSC10	23A	ST9396
PRJNA976286	SRR24861243	Carriage	NP swab	F	4	GPSC10	23A	ST9396
PRJNA976286	SRR24861242	Carriage	NP swab	M	4	GPSC321	6B	ST902
PRJNA976286	SRR24861241	Carriage	NP swab	M	4	GPSC10	23A	-
PRJNA976286	SRR24861240	Carriage	NP swab	M	4	GPSC321	6B	ST902
PRJNA976286	SRR24861238	Carriage	NP swab	F	3	GPSC10	23A	ST9396
PRJNA976286	SRR24861237	Carriage	NP swab	M	3	GPSC23	6B	ST90
PRJNA976286	SRR24861236	Carriage	NP swab	M	3	GPSC23	6B	ST90
PRJNA976286	SRR24861235	Carriage	NP swab	M	3	GPSC23	6B	ST90
PRJNA976286	SRR24861234	Carriage	NP swab	F	3	GPSC23	6B	ST90
PRJNA976286	SRR24861233	Carriage	NP swab	F	3	GPSC23	6B	ST90
PRJNA976286	SRR24861232	Carriage	NP swab	F	3	GPSC23	6B	ST90
PRJNA976286	SRR24861231	Carriage	NP swab	F	3	GPSC23	6B	ST90
PRJNA976286	SRR24861230	Carriage	NP swab	F	3	GPSC23	6B	ST90
PRJNA976286	SRR24861229	Carriage	NP swab	F	3	GPSC23	6B	ST90
PRJNA976286	SRR24861227	Carriage	NP swab	F	3	GPSC23	6B	ST90
PRJNA976286	SRR24861226	Carriage	NP swab	F	3	GPSC23	6B	ST90
PRJNA976286	SRR24861225	Carriage	NP swab	F	3	GPSC321	6B	ST902
PRJNA976286	SRR24861224	Carriage	NP swab	F	3	GPSC23	6B	ST90
PRJNA976286	SRR24861223	Carriage	NP swab	F	5	GPSC10	23A	ST9396
PRJNA976286	SRR24861222	Carriage	NP swab	F	5	GPSC1	19F	ST271
PRJNA976286	SRR24861221	Carriage	NP swab	M	6	GPSC10	23A	ST9396
PRJNA976286	SRR24861220	Carriage	NP swab	F	5	GPSC10	23A	ST9396
PRJNA976286	SRR24861219	Carriage	NP swab	F	6	GPSC10	23A	ST9396
PRJNA976286	SRR24861218	Carriage	NP swab	M	6	GPSC23	6B	ST90

Bioproject accession	SRA accession number	Disease phenotypes	Isolation source	Sex	Age (years)	GPSCs	Serotypes	MLST
PRJNA976286	SRR24861216	Carriage	NP swab	M	6	-	-	-
PRJNA976286	SRR24861215	Carriage	NP swab	M	5	GPSC14	23F	ST242
PRJNA976286	SRR24861438	Carriage	NP swab	M	5	GPSC14	23F	ST242
PRJNA976286	SRR24861437	Carriage	NP swab	F	5	GPSC1	19F	ST271
PRJNA976286	SRR24861436	Carriage	NP swab	M	6	GPSC321	6B	ST902
PRJNA976286	SRR24861435	Carriage	NP swab	F	5	-	-	-
PRJNA976286	SRR24861434	Carriage	NP swab	M	5	GPSC1	19F	ST271
PRJNA976286	SRR24861433	Carriage	NP swab	F	4	GPSC1	19F	ST271
PRJNA976286	SRR24861432	Carriage	NP swab	F	5	GPSC321	6B	ST902
PRJNA976286	SRR24861431	Carriage	NP swab	M	5	GPSC1	19F	ST271
PRJNA976286	SRR24861429	Carriage	NP swab	M	4	GPSC1	19F	ST271
PRJNA976286	SRR24861428	Carriage	NP swab	M	4	GPSC1	19F	ST271
PRJNA976286	SRR24861427	Carriage	NP swab	M	5	GPSC1	19F	ST271
PRJNA976286	SRR24861426	Carriage	NP swab	F	5	GPSC777	-	ST7502
PRJNA976286	SRR24861425	Carriage	NP swab	M	5	GPSC1	19F	ST271
PRJNA976286	SRR24861424	Carriage	NP swab	M	5	GPSC321	6B	ST902
PRJNA976286	SRR24861423	Carriage	NP swab	M	5	GPSC23	6B	ST90
PRJNA976286	SRR24861422	Carriage	NP swab	M	5	GPSC1	19F	ST271
PRJNA976286	SRR24861421	Carriage	NP swab	F	4	GPSC321	6B	ST902
PRJNA976286	SRR24861420	Carriage	NP swab	M	5	GPSC10	23A	-
PRJNA976286	SRR24861418	Carriage	NP swab	M	4	GPSC321	6B	ST902
PRJNA976286	SRR24861417	Carriage	NP swab	M	4	GPSC23	6B	ST90
PRJNA976286	SRR24861416	Carriage	NP swab	M	5	GPSC1	19F	ST271
PRJNA976286	SRR24861415	Carriage	NP swab	F	4	GPSC321	6B	ST902
PRJNA976286	SRR24861414	Carriage	NP swab	M	4	GPSC1	19F	ST271
PRJNA976286	SRR24861413	Carriage	NP swab	F	4	GPSC1	19F	ST271
PRJNA976286	SRR24861412	Carriage	NP swab	M	4	GPSC248	7C	ST2758
PRJNA976286	SRR24861411	Carriage	NP swab	M	5	GPSC1	19F	ST271
PRJNA976286	SRR24861410	Carriage	NP swab	M	5	GPSC1	19F	ST271
PRJNA976286	SRR24861409	Carriage	NP swab	F	5	GPSC14	23F	ST242
PRJNA976286	SRR24861407	NIPD	Bronchoalveolar lavage fluid	M	3	GPSC1	19F	ST271
PRJNA976286	SRR24861071	NIPD	Bronchoalveolar lavage fluid	F	1	GPSC1	19A	ST320
PRJNA976286	SRR24861070	NIPD	Bronchoalveolar lavage fluid	M	1	GPSC24	23F	ST13646

Bioproject accession	SRA accession number	Disease phenotypes	Isolation source	Sex	Age (years)	GPSCs	Serotypes	MLST
PRJNA976286	SRR24861069	NIPD	Bronchoalveolar lavage fluid	M	1	GPSC1	19F	ST14665
PRJNA976286	SRR24861068	NIPD	Bronchoalveolar lavage fluid	F	3	GPSC1	19F	ST14655
PRJNA976286	SRR24861067	NIPD	Bronchoalveolar lavage fluid	F	2	GPSC1	19F	ST271
PRJNA976286	SRR24861066	NIPD	Bronchoalveolar lavage fluid	M	4	GPSC4	14	ST876
PRJNA976286	SRR24861065	NIPD	Bronchoalveolar lavage fluid	F	3	GPSC321	6B	-
PRJNA976286	SRR24861064	NIPD	Bronchoalveolar lavage fluid	M	1	GPSC1	19F	ST271
PRJNA976286	SRR24861063	NIPD	Bronchoalveolar lavage fluid	F	0	GPSC43	2	ST4745
PRJNA976286	SRR24861061	NIPD	Bronchoalveolar lavage fluid	F	3	GPSC1	19F	ST271
PRJNA976286	SRR24861060	NIPD	Bronchoalveolar lavage fluid	F	1	GPSC16	23F	ST81
PRJNA976286	SRR24861059	NIPD	Bronchoalveolar lavage fluid	F	3	GPSC16	23F	ST81
PRJNA976286	SRR24861058	NIPD	Bronchoalveolar lavage fluid	F	0	GPSC1	19A	-
PRJNA976286	SRR24861057	NIPD	Bronchoalveolar lavage fluid	F	0	GPSC23	6B	-
PRJNA976286	SRR24861056	NIPD	Bronchoalveolar lavage fluid	M	0	GPSC1	19F	ST271
PRJNA976286	SRR24861055	NIPD	Bronchoalveolar lavage fluid	F	1	GPSC1	19F	ST271
PRJNA976286	SRR24861054	NIPD	Bronchoalveolar lavage fluid	F	2	GPSC1	19F	ST271
PRJNA976286	SRR24861053	NIPD	Bronchoalveolar lavage fluid	M	3	GPSC1	19F	ST271
PRJNA976286	SRR24861052	NIPD	Bronchoalveolar lavage fluid	M	0	GPSC16	23F	ST81
PRJNA976286	SRR24861050	NIPD	Bronchoalveolar lavage fluid	M	3	GPSC1	19F	ST271

Bioproject accession	SRA accession number	Disease phenotypes	Isolation source	Sex	Age (years)	GPSCs	Serotypes	MLST
PRJNA976286	SRR24861049	NIPD	Bronchoalveolar lavage fluid	F	3	GPSC248	7C	ST11967
PRJNA976286	SRR24861048	NIPD	Bronchoalveolar lavage fluid	F	0	GPSC248	7C	ST11967
PRJNA976286	SRR24861047	NIPD	Bronchoalveolar lavage fluid	M	1	GPSC1	19F	-
PRJNA976286	SRR24861046	NIPD	Bronchoalveolar lavage fluid	M	2	GPSC1	19F	ST271
PRJNA976286	SRR24861045	NIPD	Sputum	M	2	GPSC1	19F	ST7178
PRJNA976286	SRR24861044	NIPD	Sputum	F	0	GPSC1	19F	ST271
PRJNA976286	SRR24861043	NIPD	Sputum	M	2	GPSC1	19F	ST320
PRJNA976286	SRR24861042	NIPD	Sputum	M	0	GPSC152	15C	ST3397
PRJNA976286	SRR24861041	NIPD	Bronchoalveolar lavage fluid	F	1	GPSC321	6B	-
PRJNA976286	SRR24861597	NIPD	Bronchoalveolar lavage fluid	F	1	GPSC1	19F	ST271
PRJNA976286	SRR24861596	NIPD	Sputum	F	3	GPSC5	23A	ST338
PRJNA976286	SRR24861595	NIPD	Sputum	F	3	GPSC1	19F	-
PRJNA976286	SRR24861594	NIPD	Bronchoalveolar lavage fluid	M	0	GPSC852	6A	ST3173
PRJNA976286	SRR24861593	NIPD	Sputum	M	0	GPSC1	19F	ST271
PRJNA976286	SRR24861592	NIPD	Sputum	M	3	GPSC1	19F	ST271
PRJNA976286	SRR24861591	NIPD	Sputum	F	2	GPSC852	6A	ST3173
PRJNA976286	SRR24861590	NIPD	Sputum	M	4	GPSC1	19F	ST271
PRJNA976286	SRR24861589	NIPD	Sputum	M	4	GPSC4	14	ST876
PRJNA976286	SRR24861588	NIPD	Sputum	M	1	GPSC23	6B	ST90
PRJNA976286	SRR24861586	NIPD	Sputum	M	1	GPSC1	19F	ST4768
PRJNA976286	SRR24861585	NIPD	Sputum	F	4	GPSC1	19F	ST4768
PRJNA976286	SRR24861584	NIPD	Sputum	M	3	GPSC853	6B	ST9789
PRJNA976286	SRR24861583	NIPD	Sputum	F	0	GPSC904;9	19F	ST2097
PRJNA976286	SRR24861582	NIPD	Sputum	M	1	GPSC23	6B	ST90
PRJNA976286	SRR24861581	NIPD	Sputum	F	4	GPSC1	19F	ST4768
PRJNA976286	SRR24861580	NIPD	Sputum	M	4	GPSC69	15A	ST11972
PRJNA976286	SRR24861579	NIPD	Sputum	M	3	GPSC1	19F	ST271
PRJNA976286	SRR24861578	NIPD	Sputum	M	2	GPSC1	19F	ST236
PRJNA976286	SRR24861577	NIPD	Sputum	M	1	GPSC1	19F	ST236

Bioproject accession	SRA accession number	Disease phenotypes	Isolation source	Sex	Age (years)	GPSCs	Serotypes	MLST
PRJNA976286	SRR24861575	NIPD	Sputum	M	3	GPSC16	23F	ST81*
PRJNA976286	SRR24861574	NIPD	Bronchoalveolar lavage fluid	F	3	GPSC43	18C	ST3214
PRJNA976286	SRR24861573	NIPD	Sputum	M	3	GPSC321	6B	-
PRJNA976286	SRR24861572	NIPD	Sputum	M	1	GPSC852	6B	ST3173
PRJNA976286	SRR24861571	NIPD	Sputum	F	3	GPSC1	19F	ST271
PRJNA976286	SRR24861570	NIPD	Middle ear fluid	M	0	GPSC1	19F	ST271
PRJNA976286	SRR24861569	NIPD	Sputum	F	1	GPSC1	19F	ST271
PRJNA976286	SRR24861568	NIPD	Sputum	F	0	GPSC4	14	-
PRJNA976286	SRR24861567	NIPD	Sputum	F	0	GPSC4	14	ST876
PRJNA976286	SRR24861214	NIPD	Sputum	M	1	GPSC1	19F	ST236
PRJNA976286	SRR24861212	NIPD	Sputum	M	1	-	15A	-
PRJNA976286	SRR24861211	NIPD	Sputum	F	4	-	6C	-
PRJNA976286	SRR24861210	NIPD	Bronchoalveolar lavage fluid	M	1	GPSC10	23F	ST230
PRJNA976286	SRR24861209	NIPD	Sputum	M	3	GPSC321	6B	ST902
PRJNA976286	SRR24861208	NIPD	Sputum	M	5	GPSC321	6B	ST902
PRJNA976286	SRR24861207	NIPD	Sputum	F	1	GPSC904;9	15A	-
PRJNA976286	SRR24861206	NIPD	Sputum	M	0	GPSC853	6A	ST9789
PRJNA976286	SRR24861205	NIPD	Sputum	F	1	GPSC1	19F	ST320
PRJNA976286	SRR24861204	NIPD	Sputum	F	2	GPSC16	23F	ST81
PRJNA976286	SRR24861203	NIPD	Sputum	F	0	GPSC321	6B	ST902
PRJNA976286	SRR24861201	NIPD	Sputum	M	0	GPSC1	19A	ST320
PRJNA976286	SRR24861200	NIPD	Middle ear fluid	F	1	GPSC12	3	ST505
PRJNA976286	SRR24861199	NIPD	Sputum	M	1	GPSC852	6A	ST3173
PRJNA976286	SRR24861198	NIPD	Sputum	F	1	GPSC230	6A	-
PRJNA976286	SRR24861197	NIPD	Sputum	M	1	GPSC10	23A	-
PRJNA976286	SRR24861196	NIPD	Sputum	F	3	GPSC12	3	ST505
PRJNA976286	SRR24861195	NIPD	Sputum	M	0	GPSC1	19F	ST271
PRJNA976286	SRR24861194	NIPD	Sputum	M	11	GPSC43	9V	ST11949
PRJNA976286	SRR24861193	NIPD	Sputum	M	3	GPSC382	6B	ST7397
PRJNA976286	SRR24861192	NIPD	Sputum	M	1	GPSC321	6B	ST902
PRJNA976286	SRR24861190	NIPD	Sputum	F	0	GPSC852	6A	ST3173
PRJNA976286	SRR24861189	NIPD	Sputum	F	0	GPSC13	6A	ST473
PRJNA976286	SRR24861188	NIPD	Sputum	M	1	GPSC152	15B	ST3397
PRJNA976286	SRR24861187	NIPD	Sputum	F	1	GPSC852	6A	ST6340

Bioproject accession	SRA accession number	Disease phenotypes	Isolation source	Sex	Age (years)	GPSCs	Serotypes	MLST
PRJNA976286	SRR24861186	NIPD	Bronchoalveolar lavage fluid	M	1	GPSC152	15B	ST9765
PRJNA976286	SRR24861185	NIPD	Sputum	M	0	GPSC852	6A	ST6340
PRJNA976286	SRR24861184	NIPD	Sputum	F	1	GPSC1	19F	-
PRJNA976286	SRR24861183	NIPD	Sputum	F	2	GPSC1	19F	ST271
PRJNA976286	SRR24861406	NIPD	Sputum	M	2	GPSC1	19F	ST271
PRJNA976286	SRR24861405	NIPD	Sputum	M	3	GPSC10	23F	ST230
PRJNA976286	SRR24861403	NIPD	Sputum	M	0	GPSC1	19F	ST320
PRJNA976286	SRR24861402	NIPD	Sputum	M	0	GPSC1	19F	ST271
PRJNA976286	SRR24861401	NIPD	Bronchoalveolar lavage fluid	M	2	GPSC1	19F	ST271
PRJNA976286	SRR24861400	NIPD	Sputum	M	2	-	-	-
PRJNA976286	SRR24861399	NIPD	Sputum	F	0	GPSC852	6A	ST6340
PRJNA976286	SRR24861398	NIPD	Sputum	F	4	GPSC904;9	19F	ST2097
PRJNA976286	SRR24861397	NIPD	Sputum	M	0	GPSC321	6B	ST902
PRJNA976286	SRR24861396	NIPD	Sputum	M	5	GPSC1	19F	ST320！
PRJNA976286	SRR24861395	NIPD	Sputum	F	1	GPSC321	6B	-
PRJNA976286	SRR24861394	NIPD	Sputum	F	3	GPSC1	19F	ST271
PRJNA976286	SRR24861392	NIPD	Sputum	M	0	GPSC321	6B	ST902
PRJNA976286	SRR24861391	NIPD	Sputum	F	3	GPSC1	19F	-
PRJNA976286	SRR24861390	NIPD	Middle ear fluid	F	1	GPSC1	19F	ST271
PRJNA976286	SRR24861389	NIPD	Sputum	F	0	GPSC1	19F	ST320
PRJNA976286	SRR24861388	NIPD	Sputum	F	2	GPSC43	9V	ST280
PRJNA976286	SRR24861387	NIPD	Sputum	M	2	GPSC1	19A	ST320
PRJNA976286	SRR24861386	NIPD	Sputum	F	1	GPSC1	19F	ST320
PRJNA976286	SRR24861385	NIPD	Bronchoalveolar lavage fluid	F	3	GPSC1	19F	ST271
PRJNA976286	SRR24861384	NIPD	Sputum	M	5	GPSC248	7C	ST11967
PRJNA976286	SRR24861383	NIPD	Sputum	M	0	-	15C	-
PRJNA976286	SRR24861381	NIPD	Sputum	M	1	GPSC1	19F	ST271
PRJNA976286	SRR24861380	NIPD	Sputum	F	3	GPSC10	23F	ST230
PRJNA976286	SRR24861379	NIPD	Puncture fluid	F	0	GPSC1	19A	ST320
PRJNA976286	SRR24861378	NIPD	Sputum	F	1	GPSC1	19F	ST271
PRJNA976286	SRR24861377	NIPD	Sputum	F	1	GPSC1	19F	ST271
PRJNA976286	SRR24861376	NIPD	Middle ear fluid	F	0	GPSC1	19F	ST271
PRJNA976286	SRR24861375	NIPD	Sputum	F	2	GPSC1	19F	ST271

Bioproject accession	SRA accession number	Disease phenotypes	Isolation source	Sex	Age (years)	GPSCs	Serotypes	MLST
PRJNA976286	SRR24861039	NIPD	Middle ear fluid	F	9	GPSC852	6A	ST3173
PRJNA976286	SRR24861038	NIPD	Sputum	F	4	GPSC1	19A	ST320
PRJNA976286	SRR24861037	NIPD	Sputum	F	3	-	-	ST10236
PRJNA976286	SRR24861035	NIPD	Sputum	M	0	GPSC1	19F	ST271
PRJNA976286	SRR24861034	NIPD	Sputum	F	4	GPSC1	19F	ST271
PRJNA976286	SRR24861033	NIPD	Sputum	M	4	GPSC1	19F	ST271
PRJNA976286	SRR24861032	NIPD	Sputum	M	2	GPSC1	19F	ST320
PRJNA976286	SRR24861031	NIPD	Middle ear fluid	M	1	GPSC1	19F	ST271
PRJNA976286	SRR24861030	NIPD	Sputum	M	3	GPSC1	19F	ST271
PRJNA976286	SRR24861029	NIPD	Sputum	M	2	GPSC1	19F	ST271
PRJNA976286	SRR24861028	NIPD	Sputum	M	2	GPSC1	19F	ST320
PRJNA976286	SRR24861027	NIPD	Sputum	F	2	GPSC853	6A	ST9789
PRJNA976286	SRR24861026	NIPD	Sputum	M	1	GPSC1	19F	ST271
PRJNA976286	SRR24861023	NIPD	Sputum	F	0	GPSC152	15B	ST6555
PRJNA976286	SRR24861805	NIPD	Sputum	M	3	GPSC1	19F	ST271
PRJNA976286	SRR24861804	NIPD	Sputum	M	1	GPSC1	19F	ST320
PRJNA976286	SRR24861803	NIPD	Sputum	F	2	GPSC1	19F	ST271
PRJNA976286	SRR24861802	NIPD	Sputum	F	0	GPSC1	19F	ST271
PRJNA976286	SRR24861801	NIPD	Sputum	M	3	GPSC1	19F	ST271
PRJNA976286	SRR24861800	NIPD	Middle ear fluid	M	0	GPSC1	19F	ST271
PRJNA976286	SRR24861799	NIPD	Sputum	F	0	GPSC23	6B	ST6339
PRJNA976286	SRR24861798	NIPD	Sputum	F	0	GPSC1	19F	ST271
PRJNA976286	SRR24861797	NIPD	Sputum	M	1	GPSC1	19F	ST320!
PRJNA976286	SRR24861795	NIPD	Sputum	M	0	GPSC852	6A	ST3173
PRJNA976286	SRR24861794	NIPD	Sputum	M	1	GPSC5	23A	ST5242
PRJNA976286	SRR24861793	NIPD	Sputum	M	2	GPSC1	19F	ST271
PRJNA976286	SRR24861792	NIPD	Sputum	F	1	GPSC14	23F	ST242
PRJNA976286	SRR24861791	NIPD	Sputum	F	5	GPSC1	19F	ST271
PRJNA976286	SRR24861566	IPD	Blood	M	4	GPSC1	19A	ST320
PRJNA976286	SRR24861565	NIPD	Puncture fluid	M	7	GPSC152	15C	ST6555
PRJNA976286	SRR24861564	NIPD	Sputum	F	0	GPSC1	19F	ST271
PRJNA976286	SRR24861563	NIPD	Sputum	F	2	GPSC321	6B	ST902
PRJNA976286	SRR24861562	NIPD	Sputum	M	0	GPSC1	19F	ST271
PRJNA976286	SRR24861560	NIPD	Sputum	F	3	GPSC1	19A	ST320
PRJNA976286	SRR24861559	NIPD	Sputum	M	0	GPSC1	19F	ST271
PRJNA976286	SRR24861558	NIPD	Middle ear fluid	F	0	GPSC5	23A	ST5242
PRJNA976286	SRR24861557	NIPD	Middle ear fluid	M	0	GPSC1	19A	ST320

Bioproject accession	SRA accession number	Disease phenotypes	Isolation source	Sex	Age (years)	GPSCs	Serotypes	MLST
PRJNA976286	SRR24861556	NIPD	Middle ear fluid	M	0	GPSC1	19F	ST271
PRJNA976286	SRR24861555	NIPD	Puncture fluid	F	4	GPSC23	6B	ST90
PRJNA976286	SRR24861554	NIPD	Puncture fluid	M	7	GPSC14	23F	ST242
PRJNA976286	SRR24861553	NIPD	Puncture fluid	M	12	GPSC1	19F	ST271
PRJNA976286	SRR24861552	NIPD	Puncture fluid	M	0	GPSC1	19F	ST271
PRJNA976286	SRR24861551	IPD	Blood	F	0	GPSC1	19F	ST271
PRJNA976286	SRR24861549	IPD	Blood	F	3	GPSC1	19F	ST271
PRJNA976286	SRR24861548	NIPD	Eye secretion	M	6	GPSC186	35B	-
PRJNA976286	SRR24861547	NIPD	Bronchoalveolar lavage fluid	M	3	GPSC4	14	ST876
PRJNA976286	SRR24861546	NIPD	Sputum	F	0	GPSC13	6A	ST473
PRJNA976286	SRR24861545	NIPD	Sputum	F	1	GPSC14	23F	ST2338
PRJNA976286	SRR24861544	NIPD	Sputum	F	0	GPSC383	28F	ST3398
PRJNA976286	SRR24861543	NIPD	Sputum	M	1	GPSC1	19F	ST271
PRJNA976286	SRR24861542	NIPD	Sputum	M	1	GPSC1	19F	ST271
PRJNA976286	SRR24861541	NIPD	Middle ear fluid	F	3	GPSC4	14	ST876
PRJNA976286	SRR24861540	NIPD	Sputum	M	0	GPSC47	6B	ST386
PRJNA976286	SRR24861538	NIPD	Sputum	M	4	-	15A	ST63
PRJNA976286	SRR24861537	NIPD	Sputum	M	0	GPSC1	19F	ST320
PRJNA976286	SRR24861536	NIPD	Sputum	M	1	GPSC852	6A	ST6918
PRJNA976286	SRR24861535	NIPD	Sputum	M	0	GPSC4	14	ST876
PRJNA976286	SRR24861182	NIPD	Sputum	M	1	GPSC212	15F	ST6202
PRJNA976286	SRR24861181	NIPD	Sputum	F	0	GPSC1	19A	ST320
PRJNA976286	SRR24861180	NIPD	Sputum	M	0	GPSC1	19F	ST320
PRJNA976286	SRR24861179	NIPD	Sputum	M	0	GPSC16	23F	ST81
PRJNA976286	SRR24861178	NIPD	Sputum	M	1	GPSC4	14	-
PRJNA976286	SRR24861177	NIPD	Sputum	M	1	GPSC4	14	ST876
PRJNA976286	SRR24861175	NIPD	Sputum	F	0	GPSC16	23F	ST81
PRJNA976286	SRR24861174	NIPD	Middle ear fluid	M	1	GPSC1	19F	ST271
PRJNA976286	SRR24861173	NIPD	Sputum	M	0	GPSC23	6B	ST90
PRJNA976286	SRR24861172	NIPD	Bronchoalveolar lavage fluid	M	0	GPSC1	19F	ST271
PRJNA976286	SRR24861171	NIPD	Sputum	M	0	GPSC152	15B	ST6555*
PRJNA976286	SRR24861170	NIPD	Sputum	F	0	GPSC23	6B	ST90
PRJNA976286	SRR24861169	NIPD	Sputum	F	0	GPSC904;9	15A	ST63
PRJNA976286	SRR24861168	NIPD	Sputum	F	4	GPSC1	19F	ST271

Bioproject accession	SRA accession number	Disease phenotypes	Isolation source	Sex	Age (years)	GPSCs	Serotypes	MLST
PRJNA976286	SRR24861167	NIPD	Sputum	M	3	GPSC1	19F	ST271
PRJNA976286	SRR24861166	NIPD	Sputum	M	3	GPSC321	6B	-
PRJNA976286	SRR24861164	NIPD	Sputum	F	3	GPSC904;9	15A	ST63
PRJNA976286	SRR24861163	NIPD	Sputum	M	3	GPSC321	6B	ST902
PRJNA976286	SRR24861162	NIPD	Sputum	F	3	GPSC1	6B	-
PRJNA976286	SRR24861161	NIPD	Sputum	M	4	GPSC852	3	ST3173
PRJNA976286	SRR24861160	NIPD	Sputum	M	3	GPSC23	6B	ST90
PRJNA976286	SRR24861159	NIPD	Middle ear fluid	F	0	GPSC858	3	ST10085
PRJNA976286	SRR24861158	NIPD	Sputum	M	1	GPSC904;9	15A	ST63
PRJNA976286	SRR24861157	NIPD	Sputum	F	2	GPSC852	6A	ST3173
PRJNA976286	SRR24861156	NIPD	Sputum	F	4	GPSC158	16F	ST8250
PRJNA976286	SRR24861155	NIPD	Sputum	F	0	GPSC1	19F	ST271
PRJNA976286	SRR24861153	NIPD	Sputum	M	0	GPSC1	19F	ST271
PRJNA976286	SRR24861152	NIPD	Sputum	F	7	GPSC230	13	-
PRJNA976286	SRR24861151	NIPD	Sputum	F	6	GPSC321	6B	ST902
PRJNA976286	SRR24861374	NIPD	Sputum	M	2	GPSC43	9N	-
PRJNA976286	SRR24861373	NIPD	Sputum	F	5	GPSC1	19F	ST271
PRJNA976286	SRR24861372	NIPD	Sputum	M	4	GPSC904;9	15A	ST63
PRJNA976286	SRR24861371	NIPD	Sputum	M	3	GPSC1	19F	ST271
PRJNA976286	SRR24861370	NIPD	Sputum	F	4	GPSC321	6B	ST902*
PRJNA976286	SRR24861369	NIPD	Sputum	M	0	GPSC853	6A	-
PRJNA976286	SRR24861368	NIPD	Sputum	M	0	GPSC1	19F	ST271
PRJNA976286	SRR24861366	NIPD	Sputum	F	1	GPSC69	15A	ST11972
PRJNA976286	SRR24861365	NIPD	Sputum	M	0	GPSC23	6B	ST90
PRJNA976286	SRR24861364	NIPD	Bronchoalveolar lavage fluid	F	1	GPSC43	9N	-
PRJNA976286	SRR24861363	NIPD	Sputum	F	2	GPSC1	19F	ST271
PRJNA976286	SRR24861362	NIPD	Sputum	M	3	GPSC1	19F	ST320
PRJNA976286	SRR24861361	NIPD	Sputum	F	2	GPSC16	23F	ST81
PRJNA976286	SRR24861360	NIPD	Sputum	M	3	GPSC244	6C	ST7767*
PRJNA976286	SRR24861359	NIPD	Sputum	M	0	-	23F	-
PRJNA976286	SRR24861358	NIPD	Sputum	M	4	GPSC321	6B	ST902
PRJNA976286	SRR24861357	NIPD	Sputum	F	0	GPSC10	23A	ST9396
PRJNA976286	SRR24861355	NIPD	Sputum	F	2	GPSC152	6B	-

Bioproject accession	SRA accession number	Disease phenotypes	Isolation source	Sex	Age (years)	GPSCs	Serotypes	MLST
PRJNA976286	SRR24861354	NIPD	Sputum	F	6	GPSC69	15A	ST11972
PRJNA976286	SRR24861353	NIPD	Sputum	F	0	GPSC152	15B	ST7768
PRJNA976286	SRR24861352	NIPD	Sputum	M	10	GPSC904;9	15A	ST63
PRJNA976286	SRR24861351	NIPD	Sputum	M	0	GPSC1	19F	ST271
PRJNA976286	SRR24861350	NIPD	Sputum	M	0	GPSC23	6B	ST90
PRJNA976286	SRR24861349	NIPD	Bronchoalveolar lavage fluid	M	10	GPSC904;9	15A	ST63
PRJNA976286	SRR24861348	NIPD	Sputum	F	4	GPSC10	23F	ST230
PRJNA976286	SRR24861347	NIPD	Sputum	M	1	GPSC321	6B	ST902
PRJNA976286	SRR24861346	NIPD	Sputum	M	0	GPSC10	23F	ST230
PRJNA976286	SRR24861343	NIPD	Sputum	M	0	GPSC1	19F	-
PRJNA976286	SRR24861790	NIPD	Sputum	M	1	GPSC1	19F	ST271
PRJNA976286	SRR24861789	NIPD	Sputum	M	1	GPSC321	6B	ST902
PRJNA976286	SRR24861788	NIPD	Sputum	M	0	GPSC1	19F	ST271
PRJNA976286	SRR24861787	NIPD	Sputum	M	0	GPSC16	23F	ST81
PRJNA976286	SRR24861786	NIPD	Bronchoalveolar lavage fluid	M	1	GPSC152	15C	-
PRJNA976286	SRR24861785	NIPD	Sputum	F	1	GPSC13	6B	ST1876
PRJNA976286	SRR24861784	NIPD	Sputum	M	8	GPSC1	19F	ST271
PRJNA976286	SRR24861783	NIPD	Bronchoalveolar lavage fluid	F	6	-	14	-
PRJNA976286	SRR24861782	NIPD	Sputum	M	1	GPSC1	19F	ST271
PRJNA976286	SRR24861780	NIPD	Sputum	F	2	GPSC10	23F	ST230
PRJNA976286	SRR24861779	NIPD	Sputum	M	1	GPSC852	6A	ST3173
PRJNA976286	SRR24861778	NIPD	Sputum	M	1	GPSC158	16F	ST8250
PRJNA976286	SRR24861777	NIPD	Sputum	F	0	GPSC1	19F	ST320
PRJNA976286	SRR24861776	NIPD	Sputum	M	0	GPSC1	19F	ST271
PRJNA976286	SRR24861775	NIPD	Sputum	M	3	GPSC321	6B	ST902
PRJNA976286	SRR24861774	NIPD	Sputum	F	3	GPSC5	23A	ST338
PRJNA976286	SRR24861773	NIPD	Sputum	F	4	GPSC1	19F	-
PRJNA976286	SRR24861772	NIPD	Sputum	M	1	GPSC1	19F	-
PRJNA976286	SRR24861771	NIPD	Sputum	F	4	GPSC1	19F	ST271
PRJNA976286	SRR24861769	NIPD	Sputum	M	1	GPSC321	6B	ST902
PRJNA976286	SRR24861768	NIPD	Sputum	F	10	GPSC1	19A	ST320
PRJNA976286	SRR24861767	NIPD	Sputum	M	1	GPSC1	19F	ST320
PRJNA976286	SRR24861766	NIPD	Sputum	M	0	GPSC1	19F	ST271

Bioproject accession	SRA accession number	Disease phenotypes	Isolation source	Sex	Age (years)	GPSCs	Serotypes	MLST
PRJNA976286	SRR24861765	NIPD	Sputum	M	0	GPSC1	19A	ST320
PRJNA976286	SRR24861764	NIPD	Sputum	M	1	GPSC1	19F	ST271
PRJNA976286	SRR24861763	NIPD	Sputum	M	0	GPSC1	19F	ST271
PRJNA976286	SRR24861762	NIPD	Sputum	M	0	GPSC321	6B	ST902
PRJNA976286	SRR24861761	NIPD	Sputum	F	3	GPSC321	6B	ST902
PRJNA976286	SRR24861760	NIPD	Sputum	M	0	GPSC43	17F	ST1263
PRJNA976286	SRR24861534	NIPD	Sputum	M	3	GPSC853	6A	ST9789
PRJNA976286	SRR24861533	NIPD	Sputum	F	0	GPSC321	6B	ST902
PRJNA976286	SRR24861532	NIPD	Sputum	F	0	GPSC321	6B	ST902
PRJNA976286	SRR24861531	NIPD	Sputum	M	0	GPSC1	19F	ST271
PRJNA976286	SRR24861530	NIPD	Sputum	M	2	GPSC321	6B	ST902
PRJNA976286	SRR24861529	NIPD	Sputum	M	0	GPSC1	19F	ST271
PRJNA976286	SRR24861528	NIPD	Sputum	M	2	GPSC23	6B	ST90
PRJNA976286	SRR24861527	NIPD	Sputum	F	2	GPSC23	6B	ST90
PRJNA976286	SRR24861526	NIPD	Sputum	F	2	-	14	-
PRJNA976286	SRR24861525	NIPD	Sputum	M	3	GPSC852	6A	ST6918
PRJNA976286	SRR24861523	NIPD	Sputum	M	3	GPSC73	11A	ST99
PRJNA976286	SRR24861522	NIPD	Sputum	F	3	GPSC1	19F	ST271
PRJNA976286	SRR24861521	NIPD	Sputum	M	0	GPSC4	14	-
PRJNA976286	SRR24861520	NIPD	Sputum	M	1	GPSC321	19F	-
PRJNA976286	SRR24861519	NIPD	Sputum	F	4	GPSC321	6B	ST902
PRJNA976286	SRR24861518	NIPD	Sputum	F	0	GPSC1	19F	ST271
PRJNA976286	SRR24861517	NIPD	Sputum	M	0	GPSC1	19F	ST271
PRJNA976286	SRR24861516	NIPD	Sputum	F	1	GPSC1	19F	ST236
PRJNA976286	SRR24861515	NIPD	Sputum	M	0	GPSC1	19F	ST271
PRJNA976286	SRR24861514	NIPD	Sputum	M	3	GPSC152	15C	ST3397
PRJNA976286	SRR24861512	NIPD	Sputum	M	3	GPSC1	19F	ST271
PRJNA976286	SRR24861511	NIPD	Sputum	F	4	GPSC852	6A	ST6340
PRJNA976286	SRR24861510	NIPD	Sputum	M	3	GPSC1	19F	ST320
PRJNA976286	SRR24861509	NIPD	Sputum	M	0	GPSC852	6B	ST3173
PRJNA976286	SRR24861508	NIPD	Sputum	M	1	GPSC1	19A	ST320
PRJNA976286	SRR24861507	NIPD	Sputum	M	6	GPSC850	23F	ST1437
PRJNA976286	SRR24861506	NIPD	Sputum	F	2	GPSC1	19F	ST320
PRJNA976286	SRR24861505	NIPD	Sputum	M	1	GPSC1	19F	ST271
PRJNA976286	SRR24861504	NIPD	Sputum	M	3	-	19F	-
PRJNA976286	SRR24861503	NIPD	Sputum	M	0	GPSC1	19F	ST271
PRJNA976286	SRR24861149	NIPD	Sputum	M	1	GPSC4	14	ST876

Bioproject accession	SRA accession number	Disease phenotypes	Isolation source	Sex	Age (years)	GPSCs	Serotypes	MLST
PRJNA976286	SRR24861148	NIPD	Sputum	M	0	GPSC1	19F	ST320
PRJNA976286	SRR24861147	NIPD	Sputum	M	0	GPSC14	23F	ST880!
PRJNA976286	SRR24861146	NIPD	Sputum	F	1	GPSC321	6B	ST902
PRJNA976286	SRR24861145	NIPD	Sputum	F	3	GPSC152	15B	-
PRJNA976286	SRR24861144	NIPD	Sputum	F	1	GPSC1	19F	ST1464
PRJNA976286	SRR24861143	NIPD	Sputum	F	7	GPSC852	6A	ST3173
PRJNA976286	SRR24861142	NIPD	Sputum	M	0	GPSC5	23A	ST5242
PRJNA976286	SRR24861141	NIPD	Sputum	F	0	GPSC73	11A	ST99
PRJNA976286	SRR24861140	NIPD	Sputum	F	3	GPSC1	19F	-
PRJNA976286	SRR24861138	NIPD	Sputum	F	0	GPSC5	23A	ST5242
PRJNA976286	SRR24861137	NIPD	Sputum	M	2	GPSC1	19F	ST271
PRJNA976286	SRR24861136	NIPD	Sputum	M	0	-	19F	-
PRJNA976286	SRR24861135	NIPD	Sputum	F	4	GPSC1	19F	ST271
PRJNA976286	SRR24861134	NIPD	Sputum	M	1	GPSC1	19F	ST271
PRJNA976286	SRR24861133	NIPD	Sputum	M	0	GPSC5	23A	ST338
PRJNA976286	SRR24861132	NIPD	Sputum	F	3	GPSC321	6B	ST902
PRJNA976286	SRR24861131	NIPD	Sputum	M	0	GPSC687	39	ST6318
PRJNA976286	SRR24861130	NIPD	Sputum	F	1	GPSC10	23A	ST6227
PRJNA976286	SRR24861129	NIPD	Sputum	M	1	GPSC165	33C	ST6578
PRJNA976286	SRR24861127	NIPD	Sputum	F	3	GPSC1	19F	ST320
PRJNA976286	SRR24861126	NIPD	Sputum	M	2	GPSC1	19F	ST320
PRJNA976286	SRR24861125	NIPD	Sputum	F	2	GPSC321	6B	ST902
PRJNA976286	SRR24861124	NIPD	Sputum	F	1	GPSC1	19F	ST271
PRJNA976286	SRR24861123	NIPD	Sputum	M	0	GPSC321	6B	ST902
PRJNA976286	SRR24861122	NIPD	Sputum	F	3	-	23F	-
PRJNA976286	SRR24861121	NIPD	Sputum	M	1	GPSC1	19F	ST320
PRJNA976286	SRR24861120	NIPD	Sputum	M	2	GPSC1	19F	ST271
PRJNA976286	SRR24861119	NIPD	Sputum	M	3	GPSC1	19F	ST271
PRJNA976286	SRR24861342	NIPD	Sputum	M	0	GPSC1	19F	ST271
PRJNA976286	SRR24861340	NIPD	Sputum	M	0	GPSC16	23F	ST81
PRJNA976286	SRR24861339	NIPD	Pus	M	2	GPSC1	19A	ST320
PRJNA976286	SRR24861338	NIPD	Sputum	M	1	GPSC1	19F	ST271
PRJNA976286	SRR24861337	NIPD	Sputum	M	0	GPSC16	23F	ST81
PRJNA976286	SRR24861336	NIPD	Sputum	F	4	GPSC1	19F	ST271
PRJNA976286	SRR24861335	NIPD	Sputum	F	1	GPSC23	6A	ST90
PRJNA976286	SRR24861334	NIPD	Sputum	M	2	GPSC852	6A	ST3173
PRJNA976286	SRR24861333	IPD	Blood	F	4	-	14	-

Bioproject accession	SRA accession number	Disease phenotypes	Isolation source	Sex	Age (years)	GPSCs	Serotypes	MLST
PRJNA976286	SRR24861332	NIPD	Sputum	M	6	GPSC850	23F	ST1437
PRJNA976286	SRR24861331	NIPD	Sputum	M	1	GPSC23	6B	ST90
PRJNA976286	SRR24861328	NIPD	Sputum	F	0	-	-	-
PRJNA976286	SRR24861327	IPD	Blood	M	0	GPSC16	23F	ST81
PRJNA976286	SRR24861326	NIPD	Sputum	M	0	GPSC1	19F	ST271
PRJNA976286	SRR24861325	NIPD	Sputum	M	0	GPSC16	23F	ST81
PRJNA976286	SRR24861324	NIPD	Sputum	F	0	GPSC16	23F	ST81
PRJNA976286	SRR24861323	NIPD	Sputum	M	0	GPSC1	19F	ST271
PRJNA976286	SRR24861322	NIPD	Sputum	M	4	GPSC23	6B	ST90
PRJNA976286	SRR24861321	NIPD	Sputum	M	0	GPSC1	19F	-
PRJNA976286	SRR24861320	NIPD	Sputum	M	0	GPSC1	19F	ST271
PRJNA976286	SRR24861319	NIPD	Sputum	M	0	GPSC1	19F	ST271
PRJNA976286	SRR24861317	NIPD	Sputum	F	0	GPSC152	14	-
PRJNA976286	SRR24861316	IPD	Blood	M	2	GPSC1	19A	ST320
PRJNA976286	SRR24861315	NIPD	Sputum	M	0	GPSC1	19F	ST271
PRJNA976286	SRR24861314	NIPD	Sputum	M	0	GPSC1	19F	ST1968
PRJNA976286	SRR24861313	NIPD	Sputum	F	2	GPSC321	6B	ST902
PRJNA976286	SRR24861312	NIPD	Sputum	M	0	GPSC13	19F	ST5501
PRJNA976286	SRR24861311	NIPD	Sputum	F	3	GPSC152	15C	ST3397
PRJNA976286	SRR24861758	NIPD	Sputum	F	0	GPSC1	19F	ST271
PRJNA976286	SRR24861757	IPD	Blood	F	0	GPSC1	19F	ST271
PRJNA976286	SRR24861756	Carriage	NP swab	F	6	GPSC852	6B	ST3173
PRJNA976286	SRR24861754	Carriage	NP swab	M	5	GPSC1	19F	ST271
PRJNA976286	SRR24861753	Carriage	NP swab	M	5	GPSC16	23F	ST81
PRJNA976286	SRR24861752	Carriage	NP swab	F	4	GPSC1	19A	ST320
PRJNA976286	SRR24861751	Carriage	NP swab	F	4	GPSC1	19F	ST271
PRJNA976286	SRR24861750	Carriage	NP swab	F	4	GPSC23	6B	ST96
PRJNA976286	SRR24861749	Carriage	NP swab	F	4	GPSC16	23F	ST81
PRJNA976286	SRR24861748	Carriage	NP swab	M	4	GPSC23	6B	ST96
PRJNA976286	SRR24861747	Carriage	NP swab	F	4	GPSC5	23A	ST5242
PRJNA976286	SRR24861746	Carriage	NP swab	F	4	GPSC1	19F	-
PRJNA976286	SRR24861745	Carriage	NP swab	M	4	GPSC1	19F	ST271
PRJNA976286	SRR24861743	Carriage	NP swab	M	4	GPSC321	6B	ST902
PRJNA976286	SRR24861742	Carriage	NP swab	M	4	GPSC321	6B	ST902
PRJNA976286	SRR24861741	Carriage	NP swab	M	5	GPSC16	23F	ST81
PRJNA976286	SRR24861740	Carriage	NP swab	F	6	GPSC23	6B	ST90
PRJNA976286	SRR24861739	Carriage	NP swab	F	6	GPSC73	11A	-

Bioproject accession	SRA accession number	Disease phenotypes	Isolation source	Sex	Age (years)	GPSCs	Serotypes	MLST
PRJNA976286	SRR24861738	Carriage	NP swab	F	6	-	16F	ST6542
PRJNA976286	SRR24861737	Carriage	NP swab	M	5	-	16F	ST6542
PRJNA976286	SRR24861736	Carriage	NP swab	F	6	-	16F	ST6542
PRJNA976286	SRR24861735	Carriage	NP swab	M	5	GPSC852	6A	-
PRJNA976286	SRR24861734	Carriage	NP swab	M	4	GPSC852	6A	ST6340
PRJNA976286	SRR24861732	Carriage	NP swab	M	4	GPSC230	6B	ST3263
PRJNA976286	SRR24861731	Carriage	NP swab	F	3	GPSC321	6B	ST902
PRJNA976286	SRR24861730	Carriage	NP swab	M	4	GPSC852	6A	ST6340
PRJNA976286	SRR24861729	Carriage	NP swab	F	3	GPSC321	6B	ST902
PRJNA976286	SRR24861728	Carriage	NP swab	F	4	GPSC904;9	15A	ST374
PRJNA976286	SRR24861727	Carriage	NP swab	M	3	GPSC13	6A	ST473
PRJNA976286	SRR24861502	Carriage	NP swab	M	3	GPSC321	6B	ST902
PRJNA976286	SRR24861501	Carriage	NP swab	M	6	GPSC904;9	15A	ST63
PRJNA976286	SRR24861500	Carriage	NP swab	M	6	GPSC321	6B	ST902
PRJNA976286	SRR24861499	Carriage	NP swab	M	6	GPSC1	19F	ST320
PRJNA976286	SRR24861497	Carriage	NP swab	F	6	GPSC904;9	15A	ST63
PRJNA976286	SRR24861496	Carriage	NP swab	M	6	GPSC904;9	15A	ST374
PRJNA976286	SRR24861495	Carriage	NP swab	F	6	GPSC321	6B	ST902
PRJNA976286	SRR24861494	Carriage	NP swab	M	6	GPSC321	6B	ST902
PRJNA976286	SRR24861493	Carriage	NP swab	M	6	GPSC850	23F	ST7497
PRJNA976286	SRR24861492	Carriage	NP swab	F	4	GPSC321	6B	ST902
PRJNA976286	SRR24861491	Carriage	NP swab	F	4	GPSC321	6B	ST902
PRJNA976286	SRR24861490	Carriage	NP swab	F	4	GPSC1	19F	ST320
PRJNA976286	SRR24861489	Carriage	NP swab	F	4	GPSC321	6B	ST902
PRJNA976286	SRR24861488	Carriage	NP swab	M	5	-	-	ST10236
PRJNA976286	SRR24861486	Carriage	NP swab	M	4	GPSC73	11A	ST99
PRJNA976286	SRR24861485	Carriage	NP swab	F	4	GPSC1	19F	ST271
PRJNA976286	SRR24861484	Carriage	NP swab	M	4	GPSC321	6B	ST902
PRJNA976286	SRR24861483	Carriage	NP swab	F	5	GPSC850	23F	ST1437
PRJNA976286	SRR24861482	Carriage	NP swab	M	4	GPSC321	6B	ST902
PRJNA976286	SRR24861481	Carriage	NP swab	F	4	GPSC321	6B	ST902
PRJNA976286	SRR24861480	Carriage	NP swab	M	5	GPSC1	19F	ST271
PRJNA976286	SRR24861479	Carriage	NP swab	F	6	GPSC16	23F	-

Bioproject accession	SRA accession number	Disease phenotypes	Isolation source	Sex	Age (years)	GPSCs	Serotypes	MLST
PRJNA976286	SRR24861478	Carriage	NP swab	F	3	GPSC45	34	ST11945
PRJNA976286	SRR24861477	Carriage	NP swab	F	4	GPSC45	34	ST11945
PRJNA976286	SRR24861475	Carriage	NP swab	M	3	GPSC73	11A	ST99
PRJNA976286	SRR24861474	Carriage	NP swab	F	3	GPSC45	34	ST11945
PRJNA976286	SRR24861473	Carriage	NP swab	M	3	GPSC1	19F	ST320
PRJNA976286	SRR24861472	Carriage	NP swab	F	3	GPSC1	19F	ST320
PRJNA976286	SRR24861471	Carriage	NP swab	M	3	GPSC1	19F	ST320
PRJNA976286	SRR24861118	Carriage	NP swab	M	3	GPSC23	6B	ST90
PRJNA976286	SRR24861117	Carriage	NP swab	F	3	GPSC1	19F	ST320
PRJNA976286	SRR24861116	Carriage	NP swab	M	5	GPSC850	23F	ST7497
PRJNA976286	SRR24861115	Carriage	NP swab	F	7	GPSC850	23F	ST7497
PRJNA976286	SRR24861114	Carriage	NP swab	M	6	GPSC904;9	15A	ST63
PRJNA976286	SRR24861112	Carriage	NP swab	M	5	GPSC152	15C	ST10098
PRJNA976286	SRR24861111	Carriage	NP swab	F	5	GPSC1	19F	ST320
PRJNA976286	SRR24861110	Carriage	NP swab	M	5	GPSC152	15B	ST10098
PRJNA976286	SRR24861109	Carriage	NP swab	F	6	GPSC152	15C	ST10098
PRJNA976286	SRR24861108	Carriage	NP swab	F	4	GPSC321	6B	ST902
PRJNA976286	SRR24861107	Carriage	NP swab	M	5	GPSC73	11A	ST6739
PRJNA976286	SRR24861106	Carriage	NP swab	F	5	GPSC321	6B	ST902
PRJNA976286	SRR24861105	Carriage	NP swab	F	4	GPSC850	23F	ST7497
PRJNA976286	SRR24861104	Carriage	NP swab	M	5	GPSC1	19F	ST271
PRJNA976286	SRR24861103	Carriage	NP swab	M	4	GPSC1	19F	ST320
PRJNA976286	SRR24861101	Carriage	NP swab	M	4	GPSC1	19A	ST320
PRJNA976286	SRR24861100	Carriage	NP swab	M	5	GPSC10	23A	ST9396
PRJNA976286	SRR24861099	Carriage	NP swab	M	5	GPSC1	19F	ST320
PRJNA976286	SRR24861098	Carriage	NP swab	F	5	GPSC1	19F	ST320
PRJNA976286	SRR24861097	Carriage	NP swab	M	3	GPSC23	6B	ST90
PRJNA976286	SRR24861096	Carriage	NP swab	F	3	GPSC23	6B	ST90
PRJNA976286	SRR24861095	Carriage	NP swab	M	3	GPSC23	6B	ST90
PRJNA976286	SRR24861094	Carriage	NP swab	F	3	GPSC23	6B	ST90
PRJNA976286	SRR24861093	Carriage	NP swab	M	3	GPSC73	11A	ST99
PRJNA976286	SRR24861092	Carriage	NP swab	F	3	GPSC23	6B	ST90
PRJNA976286	SRR24861089	Carriage	NP swab	M	3	GPSC23	6B	ST90
PRJNA976286	SRR24861088	Carriage	NP swab	F	3	GPSC1	19F	ST271
PRJNA976286	SRR24861087	Carriage	NP swab	F	3	GPSC321	6B	ST902
PRJNA976286	SRR24861310	Carriage	NP swab	M	6	GPSC321	6B	ST902

Bioproject accession	SRA accession number	Disease phenotypes	Isolation source	Sex	Age (years)	GPSCs	Serotypes	MLST
PRJNA976286	SRR24861309	Carriage	NP swab	M	5	GPSC321	6B	ST902
PRJNA976286	SRR24861308	Carriage	NP swab	F	4	GPSC1	19F	ST320
PRJNA976286	SRR24861307	Carriage	NP swab	F	4	GPSC152	15B	ST3397
PRJNA976286	SRR24861306	Carriage	NP swab	F	4	GPSC152	15C	ST3397
PRJNA976286	SRR24861305	Carriage	NP swab	F	3	GPSC1	19F	ST320
PRJNA976286	SRR24861304	Carriage	NP swab	F	5	GPSC321	23F	ST902
PRJNA976286	SRR24861302	Carriage	NP swab	M	5	GPSC904;9	15A	ST63
PRJNA976286	SRR24861301	Carriage	NP swab	M	5	GPSC152	15C	ST6555
PRJNA976286	SRR24861300	Carriage	NP swab	M	6	GPSC321	6B	ST902
PRJNA976286	SRR24861299	Carriage	NP swab	M	5	GPSC321	6B	ST902
PRJNA976286	SRR24861298	Carriage	NP swab	F	6	GPSC850	23F	ST7497
PRJNA976286	SRR24861297	Carriage	NP swab	F	6	GPSC321	6B	ST902
PRJNA976286	SRR24861296	Carriage	NP swab	M	5	GPSC1	19F	ST320
PRJNA976286	SRR24861295	Carriage	NP swab	M	6	GPSC852	23F	ST3173
PRJNA976286	SRR24861294	Carriage	NP swab	F	3	GPSC321	6B	ST902
PRJNA976286	SRR24861293	Carriage	NP swab	M	5	GPSC10	23F	ST230
PRJNA976286	SRR24861291	Carriage	NP swab	M	6	GPSC904;9	15A	ST374
PRJNA976286	SRR24861290	Carriage	NP swab	F	4	GPSC69	15A	ST11972
PRJNA976286	SRR24861289	Carriage	NP swab	F	6	GPSC69	15A	ST11972
PRJNA976286	SRR24861288	Carriage	NP swab	F	5	GPSC1	19F	ST8781
PRJNA976286	SRR24861287	Carriage	NP swab	M	6	GPSC69	15A	ST11972
PRJNA976286	SRR24861286	Carriage	NP swab	M	4	GPSC321	15A	ST902
PRJNA976286	SRR24861285	Carriage	NP swab	F	3	GPSC23	6B	ST90
PRJNA976286	SRR24861284	Carriage	NP swab	F	3	GPSC23	6B	ST90
PRJNA976286	SRR24861283	Carriage	NP swab	F	4	GPSC23	6B	ST90
PRJNA976286	SRR24861282	Carriage	NP swab	F	3	GPSC23	6B	ST90
PRJNA976286	SRR24861280	Carriage	NP swab	M	4	GPSC23	6B	ST90
PRJNA976286	SRR24861279	Carriage	NP swab	M	3	GPSC23	6B	ST90
PRJNA976286	SRR24861726	Carriage	NP swab	M	3	GPSC23	6B	ST90
PRJNA976286	SRR24861725	Carriage	NP swab	M	4	GPSC23	6B	ST90
PRJNA976286	SRR24861724	Carriage	NP swab	M	4	GPSC23	6B	ST90
PRJNA976286	SRR24861723	Carriage	NP swab	M	3	GPSC23	6B	ST90
PRJNA976286	SRR24861722	Carriage	NP swab	M	4	GPSC23	6B	ST90
PRJNA976286	SRR24861721	Carriage	NP swab	M	4	GPSC23	6B	ST90
PRJNA976286	SRR24861720	Carriage	NP swab	F	4	GPSC73	11A	ST99

Bioproject accession	SRA accession number	Disease phenotypes	Isolation source	Sex	Age (years)	GPSCs	Serotypes	MLST
PRJNA976286	SRR24861719	Carriage	NP swab	F	4	GPSC16	23F	ST81
PRJNA976286	SRR24861717	Carriage	NP swab	F	3	GPSC16	23F	ST81
PRJNA976286	SRR24861716	Carriage	NP swab	M	3	GPSC158	16F	ST8250
PRJNA976286	SRR24861715	Carriage	NP swab	F	6	GPSC5	23A	ST338
PRJNA976286	SRR24861714	Carriage	NP swab	M	5	GPSC23	6B	ST90
PRJNA976286	SRR24861713	Carriage	NP swab	F	4	GPSC1	19F	ST271
PRJNA976286	SRR24861712	Carriage	NP swab	F	5	GPSC852	15A	-
PRJNA976286	SRR24861711	Carriage	NP swab	F	5	GPSC852	15A	ST3173
PRJNA976286	SRR24861710	Carriage	NP swab	M	5	-	28A	-
PRJNA976286	SRR24861709	Carriage	NP swab	F	4	GPSC852	6A	ST3173
PRJNA976286	SRR24861708	Carriage	NP swab	F	4	GPSC5	23A	ST5242
PRJNA976286	SRR24861706	Carriage	NP swab	M	5	GPSC69	15A	ST6011
PRJNA976286	SRR24861705	Carriage	NP swab	F	4	GPSC852	6A	ST3173
PRJNA976286	SRR24861704	Carriage	NP swab	F	4	GPSC852	6A	ST3173
PRJNA976286	SRR24861703	Carriage	NP swab	M	2	GPSC321	6B	-
PRJNA976286	SRR24861702	Carriage	NP swab	M	4	GPSC850	23F	ST7497
PRJNA976286	SRR24861701	Carriage	NP swab	M	4	-	11A	-
PRJNA976286	SRR24861700	Carriage	NP swab	M	3	GPSC73	11A	ST99
PRJNA976286	SRR24861699	Carriage	NP swab	M	3	GPSC73	11A	ST99
PRJNA976286	SRR24861698	Carriage	NP swab	F	4	GPSC73	11A	ST99
PRJNA976286	SRR24861697	Carriage	NP swab	F	4	GPSC850	23F	ST7497
PRJNA976286	SRR24861695	Carriage	NP swab	F	3	GPSC73	11A	ST99
PRJNA976286	SRR24861470	Carriage	NP swab	F	3	GPSC73	11A	ST99
PRJNA976286	SRR24861469	Carriage	NP swab	F	4	GPSC321	6B	ST902
PRJNA976286	SRR24861468	Carriage	NP swab	M	4	GPSC23	6B	ST90
PRJNA976286	SRR24861467	Carriage	NP swab	M	5	GPSC1	19F	ST271
PRJNA976286	SRR24861466	Carriage	NP swab	M	5	GPSC321	6B	ST902
PRJNA976286	SRR24861465	Carriage	NP swab	M	6	GPSC1	19F	ST271
PRJNA976286	SRR24861464	Carriage	NP swab	M	6	GPSC1	19F	ST271
PRJNA976286	SRR24861463	Carriage	NP swab	F	5	GPSC5	23A	ST5242
PRJNA976286	SRR24861462	Carriage	NP swab	F	4	GPSC23	6B	ST90
PRJNA976286	SRR24861460	Carriage	NP swab	M	4	GPSC1	19F	ST271
PRJNA976286	SRR24861459	Carriage	NP swab	M	4	GPSC1	19F	ST271
PRJNA976286	SRR24861458	Carriage	NP swab	F	4	GPSC69	15A	ST11972
PRJNA976286	SRR24861457	Carriage	NP swab	F	4	GPSC69	15A	ST11972
PRJNA976286	SRR24861456	Carriage	NP swab	F	3	GPSC69	15A	ST11972
PRJNA976286	SRR24861455	Carriage	NP swab	F	4	GPSC69	15A	ST11972

Bioproject accession	SRA accession number	Disease phenotypes	Isolation source	Sex	Age (years)	GPSCs	Serotypes	MLST
PRJNA976286	SRR24861454	Carriage	NP swab	M	4	GPSC1	19F	ST271
PRJNA976286	SRR24861453	Carriage	NP swab	F	3	GPSC69	15A	ST11972
PRJNA976286	SRR24861452	Carriage	NP swab	F	4	GPSC69	15A	ST11972
PRJNA976286	SRR24861451	Carriage	NP swab	F	4	GPSC69	15A	ST11972
PRJNA976286	SRR24861449	Carriage	NP swab	M	3	GPSC69	15A	ST11972
PRJNA976286	SRR24861448	Carriage	NP swab	F	4	GPSC69	15A	ST11972
PRJNA976286	SRR24861447	Carriage	NP swab	M	5	GPSC12	3	ST180

Appendix Table 2. Demographics of participants contributing *S. pneumoniae* isolates*

Feature	Infection isolates (n=349)	Carriage Isolates (n=434)	χ^2	*P*
**Gender**				
Male	202(57.9)	237(54.6)	0.84	0.359
Female	147(42.1)	197(45.4)		
**Age (years)**				
≤5	332(95.1)	371(85.5)	19.62	**<0.001**
>5	17(4.9)	63(14.5)		

*Data are presented as no. (%) or as otherwise indicated. Bold text indicates statistical significance.

Appendix Table 3. Function or pathogenic role of the 886 disease-associated k-mers (genes)

Gene	k-mer hits	-log(*p*)	*Average OR*	Protein Name	Function and/or Pathogenic Role	GO annotations
pcpA	618	18.07	1.38	Pneumococcal Choline-Binding Protein A	Protection Against Lung Infection and Sepsis	GO:0033925, GO:0008152, GO:0035821, GO:0005576
pitA	216	32.55	1.87	Pilus-2 Subunit, Ancillary Protein	Adherence	GO:0016020
pavB	210	21.02	1.28	Pneumococcal Adherence and Virulence Protein B	Adherence and Colonization	GO:0016020
pbp1A	111	58.68	2.03	penicillin-binding protein PBP1A	Antibiotic Resistance	GO:0008955, GO:0009002, GO:0008658, GO:0006508, GO:0016020
cps4D	79	9.43	1.44	Capsular Polysaccharide Biosynthesis Protein	Immune Modulation	GO:0004715, GO:0005524, GO:0045227, GO:0016310, GO:0005737

Gene	k-mer hits	-log(*p*)	*Average OR*	Protein Name	Function and/or Pathogenic Role	GO annotations
phtD	68	8.02	1.29	Pneumococcal Histidine Triad D	Adherence and Immune Evasion	-
Iga	23	52.22	1.53	IgA1 Protease	Immune Modulation	GO:0004222, GO:0008270, GO:0006508, GO:0005576, GO:0016020
zmpB	10	31.94	1.52	Zinc Metalloprotease B	Immune Evasion and Colonization	GO:0004222, GO:0008270, GO:0006508, GO:0005576, GO:0009986, GO:0016020
cpsC	9	34.27	1.56	Capsular Polysaccharide Biosynthesis Protein	Immune Modulation	GO:0005351, GO:0045227, GO:0009103, GO:0015774, GO:0005886
pbp3	9	26.22	1.47	Penicillin-Binding Protein 3	Antibiotic Resistance	GO:0009002, GO:0008360, GO:0071555, GO:0009252, GO:0006508
pspA	8	46.76	1.54	Pneumococcal Surface Protein A	Immune Modulation	GO:0046872, GO:0007155, GO:0030001, GO:0005886
lanM	7	48.02	1.56	Type 2 Lantipeptide Synthetase LanM	Toxin Production and Resistance	GO:0031179
metE	7	19.26	1.54	5-methyltetrahydropteroyltriglutamate-- homocysteine S-methyltransferase	Amino Acid Biosynthesis	GO:0003871, GO:0008270, GO:0009086, GO:0032259
cbpA	7	31.55	1.41	Choline Binding Protein A	Adherence, Immune Evasion, Colonization and Invasion	GO:0033925, GO:0008152, GO:0035821
cbpE	7	42.54	1.46	Choline Binding Protein E	Adherence	GO:0033925, GO:0008152, GO:0035821
zmpA	6	39.38	1.57	Zinc Metalloprotease A, IgA1	IgA1 Protease Enzyme and Colonization	GO:0004222, GO:0008236, GO:0008270, GO:0006508, GO:0005576, GO:0016020
zmpD	6	24.81	1.49	Zinc Metalloprotease D, IgA1 Paralog Protease	Immune Evasion and Colonization	GO:0004222, GO:0008270, GO:0006508, GO:0005576, GO:0016020
folP	6	16.25	1.42	Dihydropteroate Synthase	Biosynthesis of Cofactors, Prosthetic groups, and Carriers	GO:0004156, GO:0046872, GO:0046656, GO:0046654

Gene	k-mer hits	-log(*p*)	*Average OR*	Protein Name	Function and/or Pathogenic Role	GO annotations
pbp2B	6	59.17	1.56	Penicillin-Binding Protein 2B	Antibiotic Resistance	GO:0071972, GO:0008658, GO:0008360, GO:0071555, GO:0009252, GO:0046677, GO:0005886
secA2	5	16.73	1.38	Accessory Sec System Translocase SecA2	Protein and Peptide Secretion and Trafficking	GO:0008564, GO:0005524, GO:0065002, GO:0006605, GO:0008564, GO:0017038, GO:0005886, GO:0005737
crcB	4	19.60	1.50	Fluoride Efflux Transporter CrcB	Unknown Function	GO:1903425, GO:0005886
rrgA	4	12.41	1.44	Pilus-1 Tip Protein (Adhesin)	Adherence	GO:0016020
argH	4	47.63	1.39	Argininosuccinate Lyase	Amino Acid Biosynthesis	GO:0004056, GO:0042450, GO:0005737
srtG1	3	44.07	1.76	PI-2 pilus system class B sortase SrtG1	Adherence	GO:0016787, GO:0016020
cbpJ	3	14.53	1.54	Choline-Binding Protein J	Virulence	GO:0035821
psrP	3	23.18	1.49	Pneumococcal Serine-Rich Repeats Protein	Adherence	GO:0003677, GO:0007155, GO:0052031, GO:0044010, GO:0005576, GO:0009275, GO:0009986
srtC-1	3	31.01	1.46	Mediates host cell adhesion	Adherence	GO:0016787, GO:0016020
aph(3')-IIIa	3	27.45	1.45	aminoglycoside O-phosphotransferase APH(3')-IIIa	Amino acid biosynthesis	GO:0008910, GO:0005524, GO:0046872, GO:0016310, GO:0046677
nanA	3	18.77	1.44	Neuraminidase A	Hydrolytic Enzyme, Adherence and Colonization	GO:0052794, GO:0052795, GO:0052796, GO:0004308, GO:0009313, GO:0006689, GO:0005576, GO:0043231, GO:0005737, GO:0016020
galR	3	17.74	1.44	Galactose Operon Repressor	Regulatory Functions and DNA Interactions	GO:0000976, GO:0003700, GO:0006355
phtA	3	21.84	1.43	Pneumococcal Histidine Triad A	Adherence and Immune Evasion	GO:0008965
catA	3	17.95	1.35	Type A Chloramphenicol O-acetyltransferase	Antibiotic Resistance	GO:0008811, GO:0046677

Gene	k-mer hits	-log(*p*)	*Average OR*	Protein Name	Function and/or Pathogenic Role	GO annotations
pbp1B	3	36.59	1.54	Penicillin-Binding Protein 1B	Antibiotic Resistance	GO:0008955, GO:0009002, GO:0008658, GO:0006508, GO:0016020
pbp2X	3	42.31	1.44	Penicillin-Binding Protein 2X	Antibiotic Resistance	GO:0008658, GO:0008360, GO:0071555, GO:0009252, GO:0007049, GO:0051301, GO:0046677, GO:0005886
pspC	3	46.49	1.55	Pneumococcal Surface Protein C	Adherence, Immune Evasion, Colonization and Invasion	GO:0033925, GO:0008152, GO:0035821
mef(A)	2	30.46	1.68	Macrolide Efflux MFS Transporter Mef(A)	Antibiotic Resistance	GO:0022857, GO:0005886
rrgC	2	20.72	1.62	Pilus-1 Anchore Protein	Adherence	GO:0016020
clpX	2	45.23	1.59	ATP-dependent Clp protease ATP-binding subunit ClpX	Degradation of Proteins, Peptides, and Glycopeptides	GO:0016887, GO:0140662, GO:0016887, GO:0051603, GO:0051301, GO:0009376
pitB	2	11.59	1.58	Pilus-2 Subunit, Backbone Protein	Adherence	GO:0016020
cbpG	2	14.74	1.37	Choline-Binding Protein G	Adherence and Colonization	GO:0008236, GO:0035821, GO:0006508
gtfA	2	9.36	1.30	Accessory Sec System Glycosyltransferase GtfA	Protein Modification and Repair	GO:0016757, GO:0000166, GO:0018242, GO:0005886, GO:0005737, GO:0017122
lytA	2	7.71	1.24	Autolysin (N-Acetyl-Muramoyl-L-Alanine Amidase)	Autolytic Enzyme, Cell Wall Digestion and Autolysis	GO:0008745, GO:0030435, GO:0071555, GO:0009253, GO:0030420, GO:0035821, GO:0005576
rrgB	1	26.27	1.72	Pilus-1 Backbone Protein	Adherence	GO:0016020
fusA	1	24.15	1.63	Fructooligosaccharide ABC transporter substrate-binding protein FusA	Protein Synthesis	GO:0046872, GO:0015774, GO:0005886
msr(D)	1	22.59	1.60	ABC-F type ribosomal protection protein Msr(D)	Transport and Binding proteins,Toxin Production and Resistance	GO:0005524
srtC-2	1	28.46	1.54	PI-1 pilus system sortase SrtC-2	Adherence	GO:0016787, GO:0016020

Gene	k-mer hits	-log(*p*)	*Average OR*	Protein Name	Function and/or Pathogenic Role	GO annotations
galU	1	23.80	1.54	UTP--glucose-1-phosphate uridylyltransferase galU	Biosynthesis and degradation of surface polysaccharides and lipopolysaccharides	GO:0003983, GO:0006011, GO:0009058
mltG	1	16.52	1.47	Endolytic Transglycosylase MltG	Part of the elongasome which synthesizes peripheral peptidogylcan.	GO:0008932, GO:0071555, GO:0009252GO:0005886
gpsB	1	11.91	1.47	Cell Division Regulator GpsB	Mediates Protein Phosphorylation and Penicillin-Binding Protein Interactions	GO:0008360, GO:0007049, GO:0051301, GO:0005737
tet(M)	1	21.23	1.44	Tetracycline Resistance Ribosomal Protection Protein Tet(M)	Antibiotic Resistance	GO:0003924, GO:0005525, GO:0046677, GO:0006412
pavA	1	7.51	1.42	Pneumococcal Adherence and Virulence Protein A	Adherence, Immune Evasion, Colonization and Translocation	GO:0000049, GO:0043023, GO:0072344, GO:0005576, GO:0042603, GO:0009986, GO:0005737
ilvC	1	8.37	1.37	Ketol-acid reductoisomerase	Amino Acid Biosynthesis	GO:0004455, GO:0000287, GO:0050661, GO:0009097, GO:0009099

Appendix Table 4. List of genomes available on NCBI including those used for validation of risk prediction analyses

Disease phenotypes	Isolation source	GPSCs	Serotypes	MLST	NCBI genome accession number
IPD	Blood	GPSC544	6A	ST11918	GCA_901289825.1
IPD	Blood	GPSC141	6B	ST874	GCA_901293355.1
IPD	CSF	GPSC1	19F	ST236	GCA_001103965.1
IPD	Clinical specimen	GPSC1	19F	ST320	GCA_901286745.1
IPD	Clinical specimen	GPSC1	19A	ST320	GCA_901289905.1
IPD	Clinical specimen	GPSC1	19A	ST320	GCA_901289945.1
IPD	Clinical specimen	GPSC1	19F	ST271	GCA_901290385.1
IPD	Clinical specimen	GPSC1	19F	ST271	GCA_901290515.1
IPD	CSF	GPSC1	19F	ST236	GCA_901290595.1
IPD	Blood	GPSC1	19F	ST320	GCA_001162305.1
IPD	CSF	GPSC141	6B	ST874	GCA_901292895.1

Disease phenotypes	Isolation source	GPSCs	Serotypes	MLST	NCBI genome accession number
IPD	Blood	GPSC141	6B	ST874	GCA_901293085.1
IPD	Blood	GPSC1	19F	ST236	GCA_901293215.1
IPD	Blood	GPSC9	14	ST5285	GCA_901293445.1
IPD	Blood	GPSC1	19A	ST320	GCA_001118025.1
IPD	Blood	GPSC1	19A	ST271	GCA_001129725.1
IPD	Blood	GPSC9	14	ST63	GCA_901295225.1
IPD	Blood	GPSC213	6A	ST1988	GCA_901305565.1
IPD	Blood	GPSC185	6B	ST2016	GCA_901329145.1
IPD	Blood	GPSC1	19F	ST236	GCA_901330015.1
IPD	Blood	GPSC1	19F	ST1396	GCA_901331045.1
IPD	Blood	GPSC23	6B	ST11237	GCA_901332835.1
IPD	Clinical specimen	GPSC23	23F	ST311	GCA_901333965.1
IPD	Clinical specimen	GPSC16	23F	ST81	GCA_901334305.1
IPD	Clinical specimen	GPSC14	23F	ST242	GCA_901337455.1
IPD	Blood	GPSC1	19F	ST1421	GCA_901276655.1
IPD	Blood	GPSC6	14	ST156	GCA_901330195.1
IPD	Peritoneal fluid	GPSC1	19F	ST1421	GCA_901338195.1
IPD	Blood	GPSC1	19A	ST320	GCA_901294185.1
IPD	CSF	GPSC105	6B	ST5625	GCA_901217015.1
IPD	Pleural fluid	GPSC1	19A	ST320	GCA_901330725.1
IPD	CSF	GPSC1	19F	ST1421	GCA_901218465.1
IPD	CSF	GPSC16	23F	ST81	GCA_901252795.1
IPD	CSF	GPSC1	19F	ST1421	GCA_901273885.1
IPD	CSF	GPSC29	6A	ST11310	GCA_901293175.1
IPD	CSF	GPSC5	23F	ST338	GCA_901294975.1
IPD	CSF	GPSC1	19F	ST236	GCA_901329605.1
IPD	CSF	GPSC47	6B	ST315	GCA_901330955.1
IPD	Blood	GPSC24	6A	ST4598	GCA_901332755.1
IPD	CSF	GPSC6	23F	ST156	GCA_901338475.1
IPD	Blood	GPSC1	19F	ST271	GCA_901251715.1
IPD	Blood	GPSC1	19F	ST1421	GCA_901218775.1
IPD	Blood	GPSC1	19F	ST1421	GCA_901213735.1
IPD	Blood	GPSC1	19F	ST651	GCA_901334775.1
IPD	Pleural fluid	GPSC23	6B	ST90	GCA_901215735.1
IPD	Pleural fluid	GPSC23	6B	ST1121	GCA_901215755.1
IPD	Blood	GPSC1	19F	ST236	GCA_901315645.1
IPD	Blood	GPSC189	6B	ST5619	GCA_901252455.1
IPD	Blood	GPSC1	19F	ST651	GCA_901309215.1

Disease phenotypes	Isolation source	GPSCs	Serotypes	MLST	NCBI genome accession number
IPD	Blood	GPSC6	14	ST156	GCA_901293545.1
IPD	Blood	GPSC1	19A	ST320	GCA_901294425.1
IPD	pleural fluid	GPSC1	19A	ST320	GCA_901295055.1
IPD	Blood	GPSC1	19F	ST5459	GCA_901215195.1
IPD	Blood	GPSC1	19F	ST925	GCA_901246325.1
IPD	Blood	GPSC321	6B	ST2757	PATH2282
IPD	-	GPSC321	6B	ST902	GCA_901287305.1
IPD	-	GPSC321	6B	ST902	GCA_901305435.1
IPD	Blood	GPSC21	19F	ST347	GCA_901217095.1
IPD	Blood	GPSC21	19F	ST9930	GCA_901216415.1
IPD	Blood	GPSC21	19F	ST2715	GCA_901216645.1
NIPD	Sputum	GPSC23	6B	ST90	GCA_001101845.1
NIPD	Sputum	GPSC1	19F	ST-	GCA_001146585.1
NIPD	Sputum	GPSC1	19F	ST236	GCA_001330955.1
NIPD	Middle ear fluid	GPSC1	19F	ST320	GCA_901332285.1
NIPD	Middle ear fluid	GPSC1	19F	ST320	GCA_901332745.1
NIPD	Sputum	GPSC1	19F	ST236	GCA_001168545.1
NIPD	Middle ear fluid	GPSC1	19F	ST320	GCA_901301755.1
NIPD	Eye discharge	GPSC1	19A	ST320	GCA_901340745.1
NIPD	Middle ear fluid	GPSC1	19F	ST320	GCA_901302685.1
NIPD	Sputum	GPSC1	19F	ST236	GCA_001090545.1
NIPD	Sputum	GPSC1	19F	ST271	GCA_001990165.1
NIPD	Sputum	GPSC1	19F	ST271	GCA_001902455.1
NIPD	Middle ear fluid	GPSC1	19A	ST320	GCA_901332215.1
NIPD	Auditory canal	GPSC1	19A	ST320	GCA_001896085.1
NIPD	Sputum	GPSC1	19F	ST271	GCA_002081405.1
NIPD	Sputum	GPSC1	19F	ST271	GCA_002081315.1
NIPD	Sputum	GPSC1	19A	ST320	GCA_019399065.1
NIPD	Sputum	GPSC1	19A	ST320	GCA_019398885.1
NIPD	Sputum	GPSC1	19F	ST271	GCA_019399165.1
NIPD	Sputum	GPSC1	19F	ST271	GCA_019399085.1
NIPD	Sputum	GPSC1	19A	ST320	GCA_019397725.1
NIPD	Sputum	GPSC1	19F	ST271	GCA_019398005.1
NIPD	Sputum	GPSC1	19F	ST271	GCA_019397965.1
NIPD	Sputum	GPSC1	19F	ST271	GCA_019397925.1
NIPD	Ear canal secretions	GPSC1	19A	ST320	GCA_019397365.1
NIPD	Sputum	GPSC1	19F	ST320	GCA_019397765.1
NIPD	Sputum	GPSC1	19F	ST271	GCA_019397645.1

Disease phenotypes	Isolation source	GPSCs	Serotypes	MLST	NCBI genome accession number
NIPD	Sputum	GPSC1	19F	ST271	GCA_019397505.1
NIPD	Middle ear fluid	GPSC1	19F	ST236	GCA_001168485.1
NIPD	Sputum	GPSC1	19F	ST236	GCA_001330735.1
NIPD	Sputum	GPSC23	6B	ST90	GCA_001138625.1
NIPD	Sputum	GPSC23	6B	ST90	GCA_001132385.1
NIPD	Sputum	GPSC23	6B	ST90	GCA_001329835.1
NIPD	Middle ear fluid	GPSC23	6B	ST90	GCA_001329855.1
NIPD	Clinical specimen	GPSC23	6B	ST90	GCA_901286825.1
NIPD	Clinical specimen	GPSC23	6B	ST90	GCA_901286845.1
NIPD	Middle ear fluid	GPSC7	23F	ST9751	GCA_901246435.1
NIPD	Middle ear fluid	GPSC7	23F	ST439	GCA_901247245.1
NIPD	Middle ear fluid	GPSC7	23F	ST629	GCA_901247555.1
NIPD	Middle ear fluid	GPSC7	23F	ST629	GCA_901247755.1
NIPD	Middle ear fluid	GPSC7	23F	ST36	GCA_901332705.1
NIPD	Middle ear fluid	GPSC16	23F	ST81	GCA_901302635.1
NIPD	Sputum	GPSC230	6A	ST16328	GCA_019398925.1
NIPD	Sputum	GPSC13	6A	ST473	GCA_019398795.1
NIPD	Sputum	GPSC852	6A	ST11968	GCA_019397945.1
NIPD	Alveolar lavage fluid	GPSC852	6A	ST3173	GCA_019397425.1
NIPD	Sputum	Not assigned	19F	ST4662	GCA_002081035.1
NIPD	Middle ear fluid	GPSC1	19A	ST320	GCA_901301585.1
NIPD	Middle ear fluid	GPSC1	19A	ST320	GCA_901302985.1
NIPD	Middle ear fluid	GPSC1	19A	ST320	GCA_901301655.1
NIPD	Sputum	GPSC4	14	ST876	GCA_002081325.1
NIPD	Sputum	GPSC4	14	ST876	GCA_002081195.1
NIPD	Sputum	GPSC4	14	ST876	GCA_002081175.1
NIPD	Sputum	GPSC4	14	ST876	GCA_019397605.1
NIPD	Sputum	GPSC321	6B	ST902	GCA_019398745.1
NIPD	Sputum	GPSC321	6B	ST902	GCA_019397845.1
NIPD	Endotracheal tube tip	GPSC321	6B	ST902	GCA_019397625.1
NIPD	Middle ear fluid	GPSC32	7F	ST11901	GCA_901302105.1
NIPD	Sputum	GPSC23	6B	ST90	GCA_001173005.1
NIPD	Sputum	GPSC23	6B	ST90	GCA_900005375.1
Carriage	NP swab	GPSC141	6B	ST5293	GCA_901247035.1
Carriage	NP swab	GPSC141	6B	ST5293	GCA_901219865.1
Carriage	NP swab	GPSC116	23A	ST7707	GCA_901219685.1
Carriage	NP swab	GPSC69	15A	ST4965	GCA_901265045.1
Carriage	NP swab	GPSC23	6B	ST90	GCA_901286105.1

Disease phenotypes	Isolation source	GPSCs	Serotypes	MLST	NCBI genome accession number
Carriage	NP swab	GPSC23	6B	ST90	GCA_901286145.1
Carriage	NP swab	GPSC23	6B	ST90	GCA_901286195.1
Carriage	NP swab	GPSC23	6B	ST90	GCA_901286265.1
Carriage	NP swab	GPSC23	6B	ST90	GCA_901286295.1
Carriage	NP swab	GPSC23	6B	ST90	GCA_901286315.1
Carriage	NP swab	GPSC23	6B	ST90	GCA_901286345.1
Carriage	NP swab	GPSC23	6B	ST90	GCA_901286355.1
Carriage	NP swab	GPSC23	6B	ST90	GCA_901286365.1
Carriage	NP swab	GPSC23	6B	ST90	GCA_901286415.1
Carriage	NP swab	GPSC23	6B	ST90	GCA_901286455.1
Carriage	NP swab	GPSC165	34	ST4640	GCA_901290455.1
Carriage	NP swab	GPSC23	6B	ST90	GCA_901309365.1
Carriage	NP swab	GPSC1	19F	ST236	GCA_001110065.1
Carriage	NP swab	GPSC45	34	ST12204	GCA_901309895.1
Carriage	NP swab	GPSC1	19F	ST271	GCA_901311365.1
Carriage	NP swab	GPSC1	19F	ST1464	GCA_901311855.1
Carriage	NP swab	GPSC69	15A	ST6011	GCA_901312265.1
Carriage	NP swab	GPSC5	23A	ST5242	GCA_901312355.1
Carriage	NP swab	GPSC9	15A	ST63	GCA_901312435.1
Carriage	NP swab	GPSC16	23F	ST81	GCA_901312475.1
Carriage	NP swab	GPSC9	15A	ST63	GCA_901312655.1
Carriage	NP swab	GPSC9	15A	ST63	GCA_901313005.1
Carriage	NP swab	GPSC1	19F	ST271	GCA_901313485.1
Carriage	NP swab	GPSC1	19F	ST271	GCA_901313715.1
Carriage	NP swab	GPSC509	6B	ST9597	GCA_901220635.1
Carriage	NP swab	GPSC7	23A	ST439	GCA_901214025.1
Carriage	NP swab	GPSC16	23F	ST81	GCA_901214365.1
Carriage	NP swab	GPSC69	15A	ST5448	GCA_901214405.1
Carriage	NP swab	GPSC69	15A	ST5448	GCA_901215885.1
Carriage	NP swab	GPSC23	6B	ST90	GCA_901216465.1
Carriage	NP swab	GPSC23	6B	ST5628	GCA_901217565.1
Carriage	NP swab	GPSC23	6B	ST5628	GCA_901217935.1
Carriage	NP swab	GPSC76	6B	ST1092	GCA_901218755.1
Carriage	NP swab	GPSC1	19F	ST9906	GCA_901220035.1
Carriage	NP swab	GPSC5	23A	ST338	GCA_901253265.1
Carriage	NP swab	GPSC5	23A	ST338	GCA_901254525.1
Carriage	NP swab	GPSC23	6B	ST5628	GCA_901257675.1
Carriage	NP swab	GPSC1	19F	ST236	GCA_901260265.1

Disease phenotypes	Isolation source	GPSCs	Serotypes	MLST	NCBI genome accession number
Carriage	NP swab	GPSC1	19F	ST236	GCA_001099865.1
Carriage	NP swab	GPSC23	6B	ST7416	GCA_901261585.1
Carriage	NP swab	GPSC1	19F	ST1421	GCA_901291835.1
Carriage	NP swab	GPSC16	19F	ST81	GCA_901328025.1
Carriage	NP swab	GPSC322	34	ST7441	GCA_901329445.1
Carriage	NP swab	GPSC69	15A	ST5453	GCA_901330255.1
Carriage	NP swab	GPSC69	15A	ST5448	GCA_901337705.1
Carriage	NP swab	GPSC237	34	ST12190	GCA_901340485.1
Carriage	NP swab	GPSC1	19F	ST236	GCA_900170135.1
Carriage	NP swab	GPSC1	19F	ST236	GCA_001163425.1
Carriage	NP swab	GPSC1	19F	ST4414	GCA_001158765.1
Carriage	NP swab	GPSC1	19F	ST4414	GCA_001128505.1
Carriage	NP swab	GPSC1	19F	ST4414	GCA_001329995.1
Carriage	NP swab	GPSC1	19F	ST4414	GCA_001133245.1
Carriage	NP swab	GPSC1	19F	ST4414	GCA_001171445.1
Carriage	NP swab	GPSC1	19F	ST4414	GCA_001155585.1
Carriage	NP swab	GPSC1	19F	ST4414	GCA_001330135.1

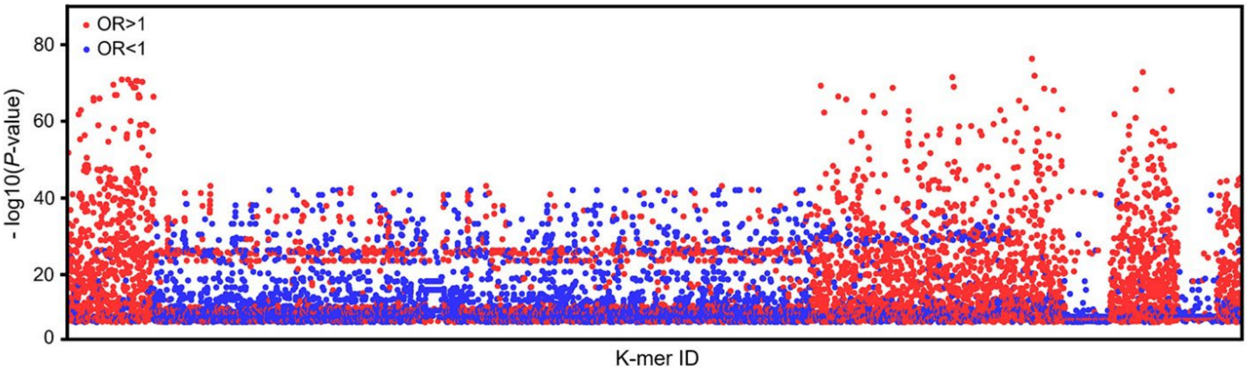

Appendix Figure 1. Manhattan plot showing statistical significance of all k-mers.

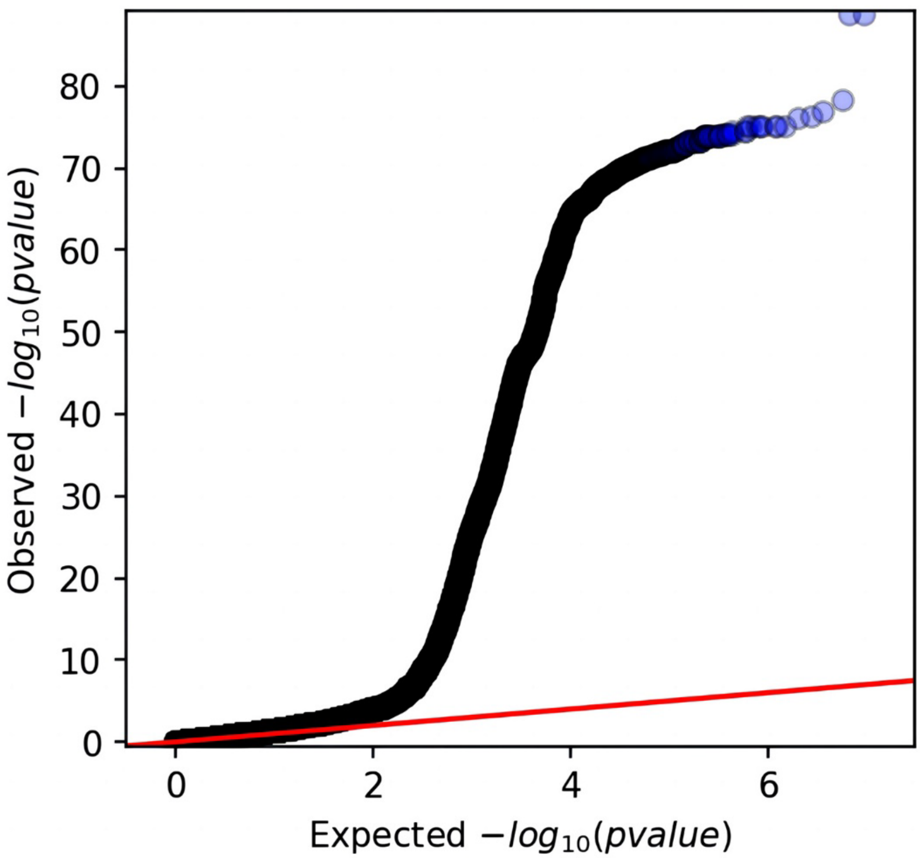

Appendix Figure 2. QQ-plot showing the expected and observed P-values for the GWAS analysis using LMM.
